# Model-based design of RNA hybridization networks implemented in living cells

**DOI:** 10.1093/nar/gkx698

**Published:** 2017-08-01

**Authors:** Guillermo Rodrigo, Satya Prakash, Shensi Shen, Eszter Majer, José-Antonio Daròs, Alfonso Jaramillo

**Affiliations:** 1 Institute of Systems and Synthetic Biology, Université d’Évry Val d’Essonne—CNRS, F-91000 Évry, France; 2 Instituto de Biología Molecular y Celular de Plantas, CSIC—Universidad Politécnica de Valencia, 46022 Valencia, Spain; 3 Warwick Integrative Synthetic Biology Centre and School of Life Sciences, University of Warwick, Coventry, CV4 7AL, UK; 4 Institute for Integrative Systems Biology (I2SysBio), University of Valencia-CSIC, 46980 Paterna, Spain

## Abstract

Synthetic gene circuits allow the behavior of living cells to be reprogrammed, and non-coding small RNAs (sRNAs) are increasingly being used as programmable regulators of gene expression. However, sRNAs (natural or synthetic) are generally used to regulate single target genes, while complex dynamic behaviors would require networks of sRNAs regulating each other. Here, we report a strategy for implementing such networks that exploits hybridization reactions carried out exclusively by multifaceted sRNAs that are both targets of and triggers for other sRNAs. These networks are ultimately coupled to the control of gene expression. We relied on a thermodynamic model of the different stable conformational states underlying this system at the nucleotide level. To test our model, we designed five different RNA hybridization networks with a linear architecture, and we implemented them in *Escherichia coli*. We validated the network architecture at the molecular level by native polyacrylamide gel electrophoresis, as well as the network function at the bacterial population and single-cell levels with a fluorescent reporter. Our results suggest that it is possible to engineer complex cellular programs based on RNA from first principles. Because these networks are mainly based on physical interactions, our designs could be expanded to other organisms as portable regulatory resources or to implement biological computations.

## INTRODUCTION

Synthetic biology offers the possibility of engineering a large variety of functional circuits *in vivo* (1–4), such as transcriptional control circuits implementing sophisticated digital behaviors (4). In this regard, RNA has also recently emerged as a substrate of choice to engineer new regulatory mechanisms, due to its high functional versatility and programmability (5–8). Natural or synthetic regulatory RNAs are now used for purposes other than the direct control of certain target genes (8,9), with the aim of implementing complex behaviors in the cell too. However, more work is needed in this direction, especially to implement cascades and feedback loops (1–4) only with RNA. To this end, we require post-transcriptional mechanisms mimicking the combinatorial action achieved by transcription factors (proteins) landing on promoter regions (DNA), as well as mechanisms to store and retrieve information through RNA molecules without the participation of DNA (i.e. RNAs with different functional states).

To address this problem, here we designed and implemented *in vivo* RNA hybridization networks, i.e. networks of RNA molecules with multiple interaction domains that can be reconfigured through hybridization events (in *trans*). Previous work on the design of synthetic regulatory RNAs *in vivo* has led to different ways of transferring information from small RNAs (sRNAs) to gene expression, such as those based on RNA regulators (5,6,8,10–15), riboswitches (16–18) and ribozymes (19–22). The use of RNA hybridization networks *in vivo* extends such works by developing RNA mediators in such information transference (RNA regulation of the RNA regulator), something instrumental to increase the regulatory power of the system and previously achieved *in vitro* (7,9,23,24).

To implement an RNA hybridization network in *Escherichia coli*, we assumed that (i) RNA–RNA interactions are initiated by non-hybridized complementary regions called toeholds (5,6); (ii) the ribosome-binding site (RBS) of a given messenger RNA (mRNA), or even the translation start site (6,13), can be considered a type of toehold mediating the interaction with the 16S ribosomal RNA; (iii) RNA-RNA interactions can simply be explained by 2D energetic features (5,25); and (*iv*) the assembly of multiple RNA strands is mostly hierarchical (7,24). We then exploited physicochemical RNA models (26–29) to predict the different stable conformational states and free energy levels of the network. Such predictability allows for the design of novel gene circuits based on RNA with sophisticated functionalities, as done with DNA strands *in vitro* (30–32).

We focused on the particular case of a linear network implemented by RNAs with two interaction domains (i.e. an RNA hybridization chain reaction) (7) and that results in the activation of gene expression (output). Initially, the conformational state of given RNA corresponds to the OFF state, where the domain to interact with an upstream RNA (trigger RNA) is active, and the domain to interact with a downstream RNA (target RNA) is inactive (*cis*-repressed). Upon interaction with the trigger RNA, the domain to interact with the target RNA becomes active (ON state). In this article, we first present a general computational model for creating RNA hybridization networks. We then present the design of five minimal networks as a proof-of-concept of the approach. We finally show several experimental results that prove that these networks are functional at the molecular and cellular levels, which validate the predictability of the model.

## MATERIALS AND METHODS

### RNA sequence design

We exploited a thermodynamic model to design cascades of regulatory RNAs to finally control gene expression. The system is composed of three different RNA species: two sRNAs and one 5′ untranslated region (UTR). We constructed an objective function based on free energies and RNA structures (Supplementary Figure S1), which were calculated thanks to a physicochemical model (26,27). In particular, this involved the energies of activation and hybridization corresponding to the interaction between the two sRNAs and the energies of activation and hybridization corresponding to the interaction between the sRNA complex and the 5′ UTR (see more details in Supplementary Information). The objective function also accounted for the degree of occlusion and exposure of the RBS within the 5′ UTR intramolecular and intermolecular structures.

We applied a Monte Carlo simulated annealing optimization algorithm (33) to perform the *de novo* sequence design. Rounds of random mutations were applied and selected with such energy-based objective function (Supplementary Figure S2), an empirical linear function that integrates all energetic contributions to the intended regulatory behavior and that must be minimized. For that, we extended a previously reported algorithm for RNA design (5,25). We used the Vienna RNA package (26) for energy and structure calculations. The sequences of the riboregulators engineered in this work, as well as their cognate 5′ UTRs, are shown in Supplementary Table S1.

### Plasmid construction

The different sRNA systems were chemically synthesized (IDT) and cloned in a pSTC2-based plasmid that contained a pSC101m replication origin (a mutated pSC101 ori giving a high copy number; E93K in *repA*) and a kanamycin resistance marker (Supplementary Figure S3). The pSTC2 vector, used in our previous works (22), has a superfolder green fluorescent protein (sfGFP) (34) as reporter gene, with a *ssr*A degradation tag (35) for fast turnover. The promoters P_LlacO1_ and P_LtetO1_ (36) control the expression of the two sRNAs, whereas the mRNA (containing the 5′ UTR) is constitutively expressed from promoter J23119. Strains and plasmids used in this study are listed in Supplementary Table S2.

### Cell culture and reagents

*Escherichia coli* strain DH5α (Invitrogen) was used for plasmid construction purposes as described in the manual (37). Characterization experiments were performed in *E. coli* DH5α-Z1 cells (Clontech) or in *E. coli* K-12 MG1655-Z1 cells (both *lacI*^+^*tetR*^+^) for control over the promoters P_LlacO1_ and P_LtetO1_ (the Z1 cassette produces LacI and TetR proteins (36)). As external inducers, we used isopropyl-β-D-thiogalactopyranoside (IPTG) and anhydrotetracycline (aTc). For characterization in a fluorometer (TECAN) or in a flow cytometer, plasmids carrying systems trigR11, trigR1 and trigR2 were transformed into DH5α-Z1 cells, while plasmids carrying systems trigR31 and trigR32 were transformed into MG1655-Z1 cells. Moreover, the plasmid carrying system trigR2 was transformed into MG1655-Z1 cells for characterization in a microfluidic device.

Cells were grown aerobically in LB or in M9 minimal media, prepared with M9 salts (Sigma-Aldrich), glycerol (0.8%, vol/vol) as the only carbon source, CaCl_2_ (100 μM), MgSO_4_ (2 mM), and FeSO_4_ (100 μM). The kanamycin concentration was 50 μg/mL. Cultures were grown overnight at 37°C and at 225 rpm from single-colony isolates before being diluted for *in vivo* characterization. 1 mM IPTG (Thermo Scientific) was used for full activation of promoter P_LlacO1_ when needed, and 100 ng/ml aTc (Sigma-Aldrich) was used for full activation of promoter P_LtetO1_. For microfluidic cultures, cells were grown aerobically in fresh LB and in LB supplemented with 0.05% sulforhodamine B (Sigma-Aldrich), and IPTG + aTc (i.e. we used sulforhodamine B to monitor the presence of inducers in the chamber) (22).

### *In vitro* RNA–RNA interaction

To perform the *in vitro* transcription, 3 μg of each pUC18-derived plasmid (see details in Supplementary Information) was digested with Eco31I, and purified with silica-based columns (Zymo). We used approximately 1 μg of digested plasmid in the reaction. This was in 20 μl: 10 μl of plasmid, 2 μl buffer 10× (Roche), 0.4 μl DTT 10 mM, 1 μl NTPs 10 mM (Thermo Scientific), 0.5 μl Ribolock (40 U/μl, Thermo Scientific), 1 μl inorganic pyrophosphatase (0.1 U/μl, Thermo Scientific), 1 μl T7 RNA polymerase (50 U/μl, Epicentre) and 4.1 μl H_2_O. We incubated the mix for 1 h at 37°C, and then added 20 μl of loading buffer with formamide. The samples were heated at 95°C for 1.5 min, then cooled on ice, and then separated by electrophoresis (200 V, 2.5 h) in a 10% polyacrylamide gel, containing 8 M urea, TBE (1×). We cut the bands corresponding to the full-length RNAs for purification. The presence of RNA was confirmed by loading a small part of the purified preparations in another polyacrylamide gel.

For the reaction of RNA–RNA interaction, we used ∼20 ng of RNA for each of the transcripts. The buffer of the reaction was 50 mM Tris–HCl pH 7.5, 10 mM MgCl_2_, 20 mM NaCl. The mix (20 μL) was denatured (1.5 min at 95°C) and slowly cooled (15 min at room temperature) (38). We then added 1.5 μl glycerol (87%) and 0.2 μl bromophenol blue–xylene cyanol (100×) to load the gel (15% polyacrylamide, buffer TAE, 1 mm thick), which was run for 2 h at 75 mA at 4°C. The gel was stained first with ethidium bromide and then with AgNO_3_. We used the DNA molecular weight marker XIII (50 bp ladder, Roche).

ImageJ was used to quantify the bands (39), which are assumed to be proportional to mass. The apparent dissociation constants were calculated by translating the mass fractions into molar fractions with the molecular weight of the RNAs (see details in Supplementary Information).

### Fluorescence quantification

Cells were grown overnight in LB medium and were then refreshed by diluting 1:200 in M9 medium. They were grown for additional 2 h to then load 200 ml in each well of the plate (Custom Corning Costar). Appropriate inducers (none, aTc, IPTG, or aTc + IPTG) were introduced when needed during refreshing. The plate was incubated in an Infinite F500 multi-well fluorometer (TECAN) at 37°C with shaking. It was assayed with an automatic repeating protocol of absorbance measurements (600 nm absorbance filter) and fluorescence measurements (465/35 nm excitation filter—530/25 nm emission filter for sfGFP) every 15 min. All samples were replicated on the plate from three different colonies.

Normalized fluorescence was obtained by subtracting the background values corresponding to M9 medium (in both fluorescence and absorbance values) and then dividing fluorescence by absorbance at OD_600_ ≈ 0.5 (22). Corrected normalized fluorescence was obtained by subtracting the fluorescence of plain cells (autofluorescence).

### Single-cell microfluidic analysis

The design of our microfluidic device was performed in AUTOCAD (AUTODESK), and it was already applied to study a synthetic genetic oscillator (40). All images were acquired using Zeiss Axio Observer Z1 microscopy (Zeiss). The microscope resolution was 0.24 μm with Optovariation 1.6×, resulting total magnification 1600× for both bright field and fluorescent images. Images were analyzed with MATLAB (MathWorks). Cells were tracked by defining a cell-to-cell distance matrix and the cell lineages were reconstructed. Finally, the fluorescence level of each cell in each fluorescence frame was extracted (see Supplementary Figure S4 for the setup).

### Flow cytometry analysis

Cells were grown overnight in LB medium and were then diluted 1:200 in fresh LB medium containing inducers (none, aTc, IPTG, or aTc + IPTG) and incubated to reach an OD_600_ of 0.2–0.4. Afterward, cells were diluted again in 1 ml PBS. All expression data were analyzed using a Becton-Dickinson FACScan flow cytometer with a 488 nm argon laser for excitation and a 530/30 nm emission filter (sfGFP). Gene expression of each sample was obtained by measuring the fluorescence intensity of thousands of cells. The data were analyzed using the Cytobank webserver by gating the events using scatter ranges, and then fluorescence histograms (without subtracting autofluorescence) plotted with MATLAB.

## RESULTS

### Thermodynamic model of RNA hybridization networks

**Figure 1.**
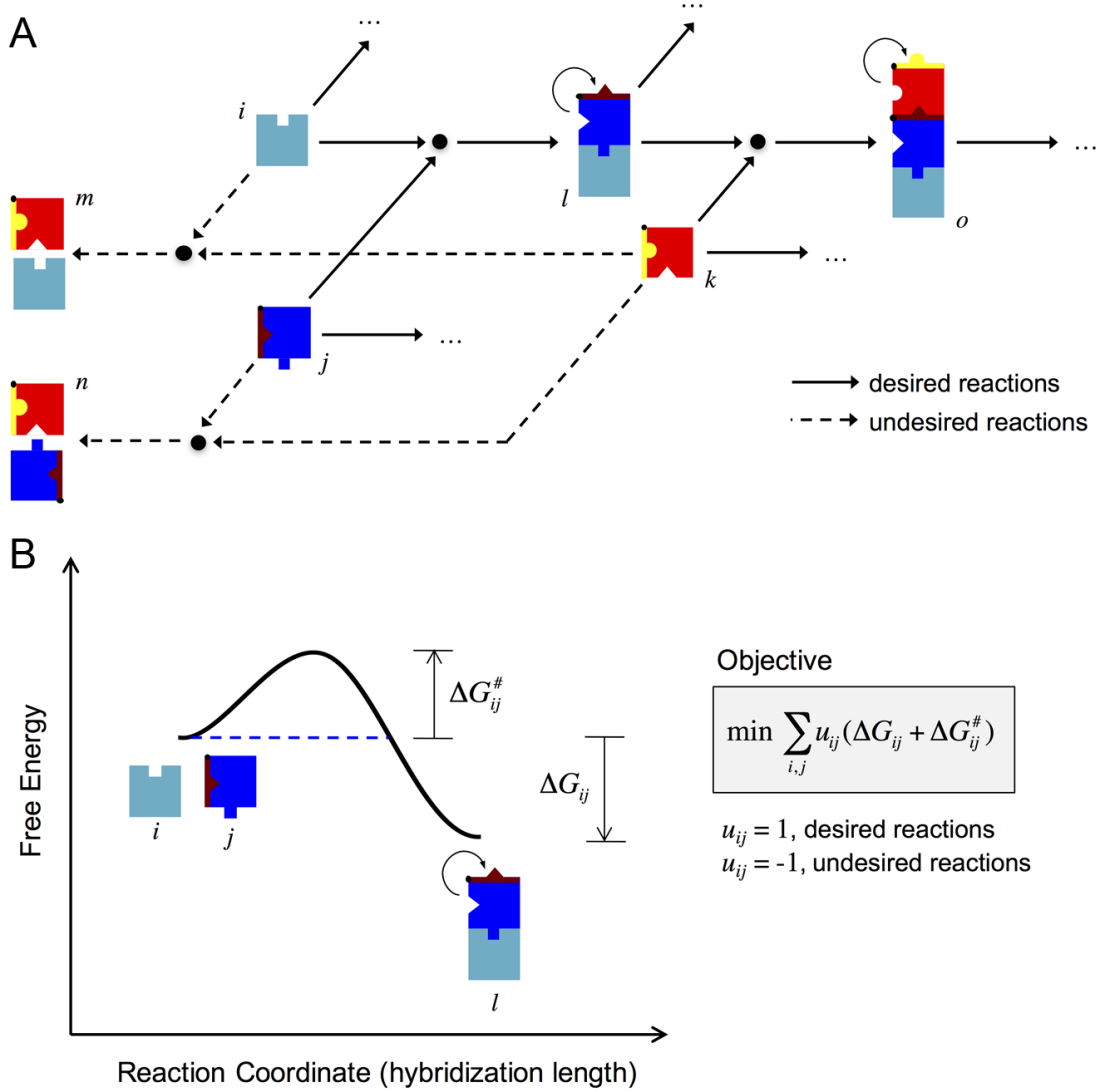
(**A**) General scheme of an RNA hybridization network implemented with RNA-triggered riboregulators (i.e., riboregulators that allosterically switch from an OFF state to an ON state upon interaction with another riboregulator; colored boxes with notches). The arrows indicate the possible hybridization reactions; solid lines for desired interactions (energetically favorable) and dashed lines for undesired interactions (energetically unfavorable). (**B**) Energy landscape of a particular reaction within the network (between the molecules *i* and *j*). This shows the different conformational states and their free energy levels as a function of a reaction coordinate (number of intermolecular base pairs). A general objective function, which should be minimized, is shown. The terms Δ*G_ij_* and Δ*G^#^_ij_* correspond to the free energies of hybridization and activation, respectively. Note that the free energy of hybridization is a negative magnitude, whereas the free energy of activation is a positive magnitude.

We built a coarse-grained model, based on energies and structures, to describe the dynamic behavior a network consisting of an arbitrary number of different RNA molecules that can interact with each other (Figure 1A). Each node in the network is an individual species or a complex. The energy landscape associated with a given interaction (between nodes *i* and *j*) is shown in Figure 1B. The reaction coordinate was defined as the number of intermolecular hydrogen bonds (or base pairs) between the two RNA molecules. In the energy landscape, one barrier (the free energy of activation; Δ*G^#^_ij_*) impinges on the progression of the reaction (41). This is associated with the degree of exposure of the toeholds to the solvent, and it has to be low to permit the initiation of the reaction (kinetic aspect). In addition, for an efficient reaction, the free energy of hybridization (Δ*G_ij_*) has to be as negative as possible to ensure irreversibility in the intermolecular interaction (this represents the thermodynamic aspect of the reaction).

Following this model, it is possible to design a given RNA hybridization network by specifying a set of desired and undesired interactions. The nucleotide sequences of the different RNAs can be obtained by minimizing/maximizing the objective free energy (Δ*G_ij_* + Δ*G^#^_ij_*) of desired/undesired interactions (Supplementary Figure S1). Consequently, we developed a computational workflow to automate the network design process (Supplementary Figure S2), although a rational design could also be possible. In particular, we applied heuristic optimization (5) using Vienna RNA (26).

### Design of RNA hybridization networks coupled to gene expression

**Figure 2.**
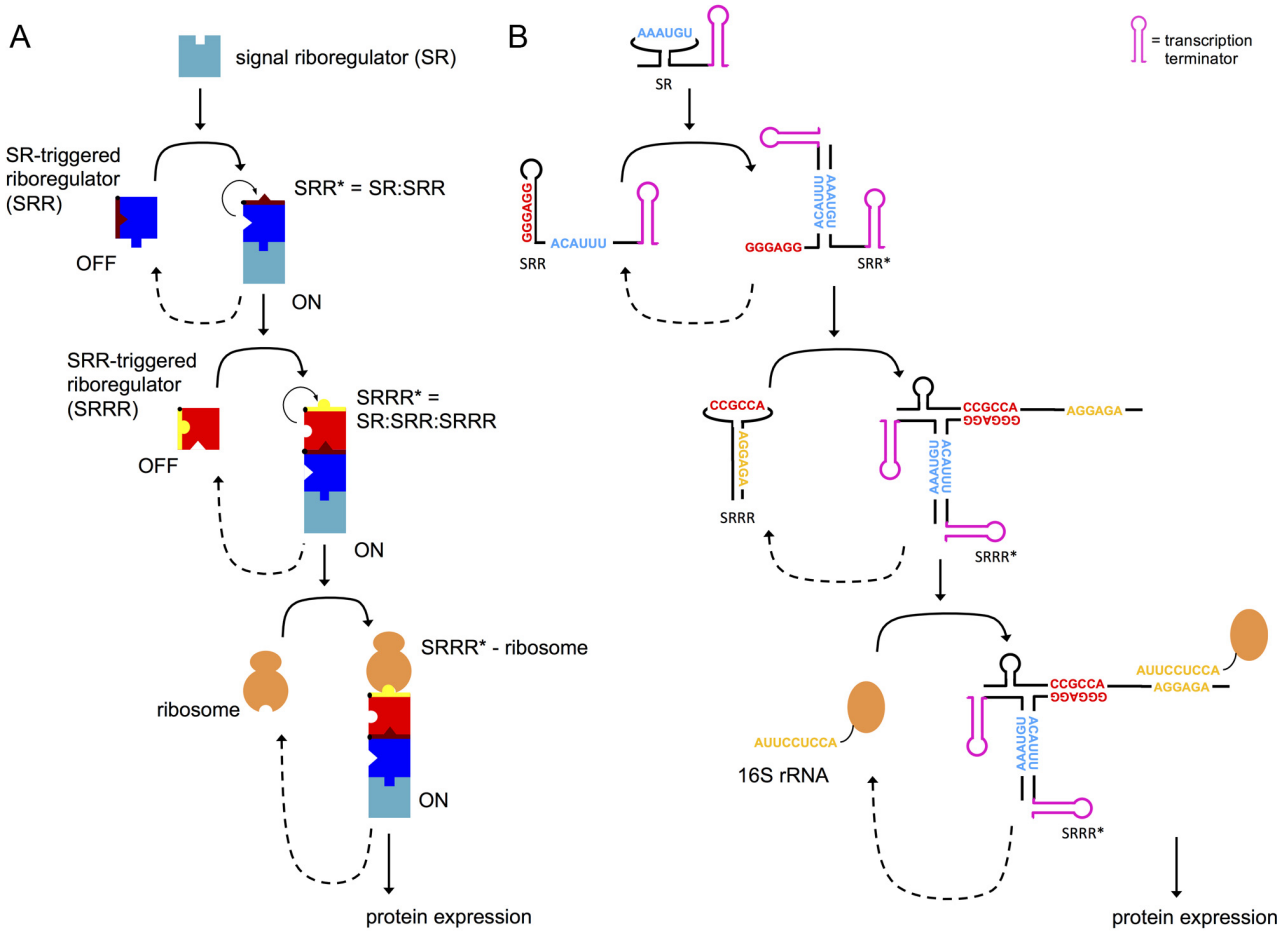
(**A**) Scheme of the simplest theoretical form of an RNA hybridization network, a chain reaction. (**B**) Sequence-structure schematics of a designer chain reaction (system trigR2). The toehold sequence for the interaction between the two sRNAs (SR and SRR) is shown in blue, and the toehold sequence for the interaction between the heterodimer (sRNA complex) and the 5′ UTR (SRRR) is shown in red. In the 5′ UTR, the RBS (shown in yellow) functions as a kind of toehold to interact with the ribosome.

We applied this thermodynamic model to guide the design of a simple network consisting in a chain reaction of three RNA molecules, together with the ribosome, as a proof-of-concept. Figure 2A illustrates this cascade (see also Supplementary Figure S5 where we detail the corresponding energy landscape). The first molecule is an sRNA that we call a signal riboregulator (SR). This molecule can interact with a second molecule, another sRNA called an SR-triggered riboregulator (SRR), which is initially in the OFF state (i.e., with a hidden/inactive toehold to interact with the downstream element). The resulting complex (SRR*), which is in the ON state (i.e. with the aforementioned toehold exposed/active), can subsequently interact with a third molecule, called an SRR-triggered riboregulator (SRRR). This strategy could facilitate the creation of larger cascades at the post-transcriptional level. For the purpose of designing a network to control gene expression, we considered SRRR to be the expression platform, i.e., a *cis*-repressed 5′ UTR of a given mRNA (see Supplementary Information for a rationale about the interaction with the ribosome).

By calculating the objective free energy (Δ*G_ij_* + Δ*G^#^_ij_*) for each interaction, we can evaluate if a set of three arbitrary RNA molecules follows the energetic/structural requirements. In this work, we designed five different riboregulatory cascades: trigR31, trigR32, trigR1, trigR2, and trigR11 (see complete sequences in Supplementary Table S1). Figure 2B illustrates the implementation of system trigR2. One of the toeholds that nucleates the interaction between SRR* and SRRR is hidden within the unhybridized structure of SRR. However, both toeholds that nucleate the interaction between SR and SRR are exposed (active) within their respective unhybridized structures. This ensures that the hybridization reaction between SR and SRR can occur. As a result, within the hybridized structure of SRR*, the toehold that nucleates the interaction with SRRR becomes active. Supplementary Figure S6 shows the sequence-structure schematics of all these systems (only the toehold sequences are shown for simplicity), where different interaction modes can be identified (i.e. different toehold positions and different intermolecular complex structures).

We followed different strategies to obtain the sequences implementing these systems using the same thermodynamic model. Systems trigR31 and trigR32 were obtained by sequential design, i.e. first designing the sequences of SR and SRR, and then the sequence of SRRR. The sequences of SR and SRR of these systems were based on previous riboregulatory elements taken from (6) (see sequence design details in Supplementary Information). Then, we computationally designed the corresponding SRRRs. By contrast, systems trigR1 and trigR2 were obtained by full design, i.e., designing the sequences of SR, SRR and SRRR at the same time. System trigR1 was obtained by specifying the unhybridized structures of SR and SRR, while for system trigR2, the hybridized structure of SRR* was specified (introduced, in both cases, as sub-objectives in the global objective function and not as enforced constraints). These specifications, although not functionally required, were introduced to prevent premature degradation of unstructured sRNAs. Finally, the sequences of system trigR11 were based on our previously published riboregulatory system RAJ11 (5). We split the sRNA into two halves (SR and SRR), and considered the cognate 5′ UTR as SRRR (Supplementary Figure S7). This resulted in a system based on the formation of a three-way junction (see sequence design details in Supplementary Information).

Because the sequences were selected only according to energetic criteria, the designed systems present different implementations in terms of toehold position within the structures. Indeed, we recognized active toeholds in the unpaired 5′ end (SRR of trigR31 and trigR32; SRRR of trigR31), in a loop (SR of trigR1, trigR2 and trigR11; SRR of trigR1 and trigR11; SRRR of trigR32, trigR1, trigR2 and trigR11), and in an inter-stem space (SR of trigR31 and trigR2; SRR of trigR2). This stresses the high designability of RNA hybridization networks.

### Characterization of RNA hybridization networks at the population level

**Figure 3.**
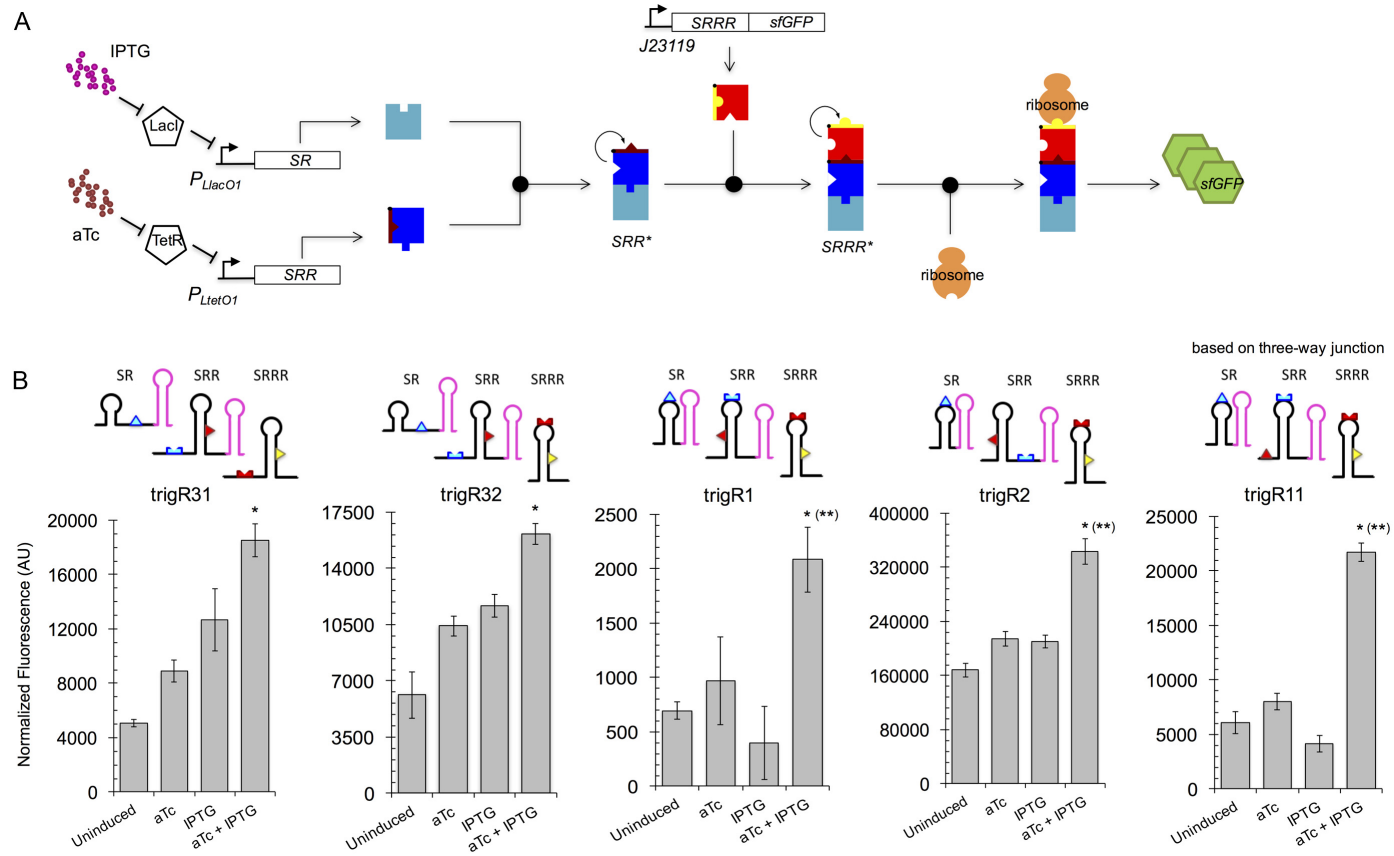
Functional characterization of designer RNA hybridization networks in bacterial cell populations. (**A**) Scheme of the engineered sRNA circuit. Promoters P_LlacO1_ and P_LtetO1_ control the expression of the two sRNAs (SR and SRR), which can be tuned with different concentrations of the external inducers IPTG and aTc, whereas the mRNA (SRRR:sfGFP) is constitutively expressed from promoter J23119. The two sRNAs first interact to form a complex that is then able to activate a *cis*-repressed gene. The reporter gene encodes a sfGFP. (B) Fluorescence results (arbitrary units, AU) from the sRNA systems trigR31, trigR32, trigR1, trigR2 and trigR11 for all possible combinations of inducers. Error bars represent standard deviations over three biological replicates. The structural schemes of each single species implementing a system are shown. In each case, the asterisk (or two asterisks in brackets) denotes *P* < 0.05, one-tailed Welch *t*-test, comparing the fluorescence level when both inducers are present with respect to the level when there is only one inducer (or the level reached by the additive effect of the two inducers).

To test the functionality of our computational designs *in vivo*, the RNA systems were implemented as separate transcriptional units (with their respective promoters and terminators) in plasmids (Supplementary Figure S3, Table S2). These were then electroporated into *E. coli* cells expressing the transcriptional repressors LacI and TetR (see Materials and Methods; Figure 3A illustrates the engineered RNA circuit). We used P_L_-based inducible promoters (36) for controlling the expression of SR and SRR with the external inducers isopropyl-β-d-thiogalactopyranoside (IPTG) and anhydrotetracycline (aTc), respectively. We used a superfolder green fluorescent protein (sfGFP) (34) with a degradation tag as the output for the circuits, because its fast maturation and degradation allows a better correlation between fluorescence and gene expression (especially in time-dependent experiments). Figure 3B shows the dynamic ranges (characterized by bulk fluorometry) of the engineered systems, probing the regulation of gene expression in living cells with two interacting sRNAs, as well as the versatility of the toeholds within different structural contexts. We also observed that the expression platforms (SRRRs) in systems trigR11 and trigR1 exert a much tighter control of the OFF state than those in the other systems (trigR2 being the one with the highest expression levels). Subsequently, we assessed the statistical significance of the reported activation folds. We found, for all systems, that the increase in fluorescence in response to both inducers (leading to the formation of complex SRRR*) is significantly greater than the increase in fluorescence induced by either IPTG or aTc individually (one-tailed Welch *t*-test, *P* < 0.05; Figure 3B). We also found, for systems trigR11, trigR1, and trigR2, that the sum of individual increases in fluorescence with IPTG and aTc is significantly smaller than the increase with both inducers (one-tailed Welch *t*-test, *P* < 0.05; Figure 3B). We thus confirmed the model-based designability of RNA hybridization networks.

In addition, we investigated the effects of some of the attributes defining the molecular implementation of the systems. In terms of stationary behavior, it is expected that the stability of the output protein does not modify substantially the activation fold (42). We characterized system trigR11 with a stable and unstable sfGFP, as this system shows one of the lowest expression levels, obtaining a slightly higher dynamic range with the more stable variant (Supplementary Figure S8). Also, the formation of the trimeric complex (SRRR*), and then sfGFP expression, greatly depends on the strength of the promoters that express the RNAs (43), as the dissociation constants between synthetic RNAs that hybridize are high (see below). We characterized the graded response of system trigR2 with IPTG and aTc, showing this dependence (Supplementary Figure S9). Finally, synthetic RNAs do not exploit the intricate cellular machinery. We introduced an Hfq target site in SRR (sequence MicF-M7.4 from (44)) to ask if the activation fold would be higher, as this RNA chaperone has a key role in post-transcriptional regulation (45). Using the system trigR31, as it exhibits the less digital behavior, we did not find an enhancement (Supplementary Figure S10A; see Supplementary Information for a rationale).

### Probing the orthogonality between RNA-triggered riboregulators

**Figure 4.**
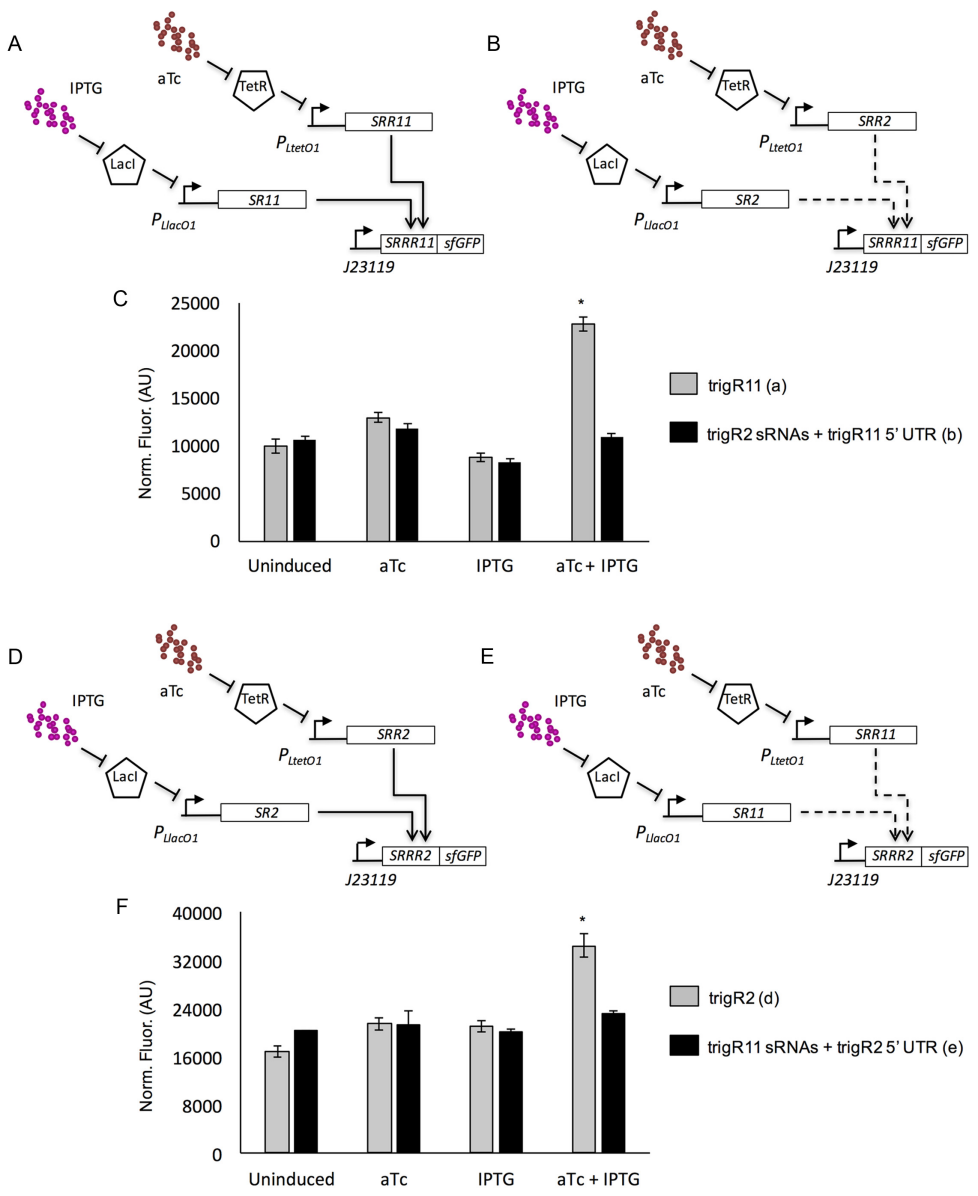
Characterization of the orthogonality of two designer RNA hybridization networks (trigR11 and trigR2) in bacterial cell populations. (**A**) Scheme of the system trigR11 (with cognate sRNAs and 5′ UTR). (**B**) Scheme of a crossed system with non-cognate elements, where the sRNAs correspond to system trigR2 and the 5′ UTR corresponds to system trigR11. Promoters P_LlacO1_ and P_LtetO1_ control the expression of the two sRNAs (SR and SRR), which can be tuned with external inducers IPTG and aTc, whereas the mRNA (SRRR:sfGFP) is constitutively expressed from promoter J23119. (**C**) Fluorescence results (arbitrary units, AU) from the systems shown in (A) and (B). Error bars represent standard deviations over three biological replicates. (**D**) Scheme of the system trigR2 (with cognate sRNAs and 5′ UTR). (**E**) Scheme of a crossed system with non-cognate elements, where the sRNAs correspond to system trigR11 and the 5′ UTR corresponds to system trigR2. (**F**) Fluorescence results (arbitrary units, AU) from the systems shown in (D) and (E). Three biological replicates. In both cases, the asterisk denotes *P* < 0.05, one-tailed Welch *t*-test, comparing the fluorescence level for the cognate pair with respect to the level for the non-cognate pair.

Next, we performed an experimental study to assess the specificity of our designed sRNAs, using the systems trigR11 and trigR2 (Figure 4A and D). We chose these two systems because they seem to have the highest expression levels, what might favor a problem of cross-regulation when both systems work in the same cell. For this analysis, we constructed two new, crossed systems: one with the sRNAs (SR and SRR) from trigR2 and the 5′ UTR (SRRR) from trigR11 (Figure 4B), and the other with the sRNAs from trigR11 and the 5′ UTR from trigR2 (Figure 4E). The same promoters were used (P_L_-based inducible promoters for the sRNAs, and a constitutive promoter for the mRNA). Computational simulations of cofolding using Vienna RNA (26) indicated that there is no significant free energy gap to promote hybridization between SRR* and SRRR if they are non-cognate pairs. When we tested this experimentally, we found significant activation of sfGFP in the presence of both IPTG and aTc only for cognate pairs (one-tailed Welch *t*-test, *P* < 0.05; Figure 4C and F). These results suggest that different RNA hybridization networks can be deployed in the same cell.

### Characterization of RNA hybridization networks at the single-cell level

**Figure 5.**
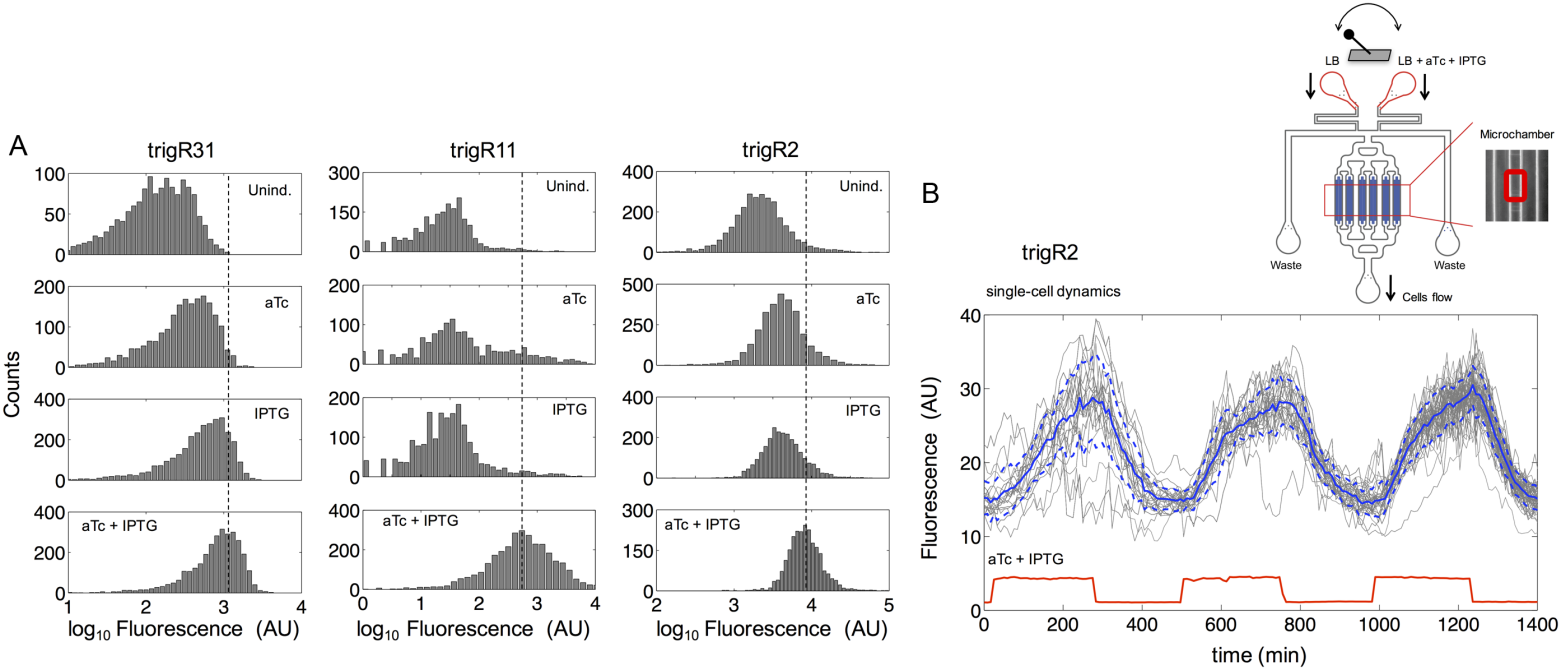
Functional characterization of designer RNA hybridization networks in single bacterial cells. (**A**) Fluorescence distributions of multiple individual cells obtained by flow cytometry for systems trigR31, trigR11 and trigR2. Unind., uninduced. (**B**) Dynamic single-cell tracking of fluorescence (arbitrary units, AU) in one microchamber of the microfluidics device under time-dependent induction with IPTG and aTc for system trigR2 (∼100 cells). Both inducers were applied with a period of 8 h (i.e. 4 h induction/ON and 4 h relaxation/OFF; square wave). The solid and dashed lines (in blue) correspond to the mean and plus/minus the standard deviation for the entire cell population, respectively. Sulforhodamine B (red fluorescent dye) was used to monitor the inducer time-dependent profile (in red). A scheme of the device is shown at the top of the panel. Bacterial cells are trapped in the microchambers (zoomed in) and exposed to a continuous flow of media, either LB or LB with inducers (switching controlled with pumps).

We then decided to study the dynamic behavior of our computational designs in single *E. coli* cells, as this would reveal to what extent the response is homogeneous (5,6,10). Flow cytometry experiments revealed significant bacterial population shift in response to both inducers (Mann–Whitney *U*-test, *P* < 0.05; Figure 5A; results for systems trigR31, trigR11 and trigR2). The reported dynamic ranges at the single-cell level are similar to those measured for the whole population (Supplementary Figure S11A). These results showed that each individual cell responds to the inducers in a relatively homogeneous manner (unimodal distributions). The observed cell-to-cell variability in output protein expression is comparable to previous scenarios of simple riboregulation in the cases of trigR31 and trigR2 (5,6,10), but system trigR11 presents larger spread (Supplementary Figure S11B).

To further explore the cell-to-cell variability during the induction dynamics of the systems, we performed a time-dependent characterization of system trigR2 using microfluidic lab-on-chip devices (Supplementary Figure S4) (3,40). This allowed us to monitor sfGFP expression in individual cells stimulated with a varying concentration of both inducers (Figure 5B). A square wave of IPTG and aTc with a period of 8 h was applied (i.e. 4 h induction/ON and 4 h relaxation/OFF), which stimulated increases and decreases of fluorescence in response (three pulses are shown in Figure 5B). We observed a delay of 25 min in the rise of fluorescence with respect to the rise of the inducers, probably due to the time required to accumulate enough RNAs. In addition, we observed certain homogenization in gene expression levels over time, as the level of noise (here, cell-to-cell variability) was much higher in the first pulse (coefficient of variation at maximal expression, CV = 21%) than in the third pulse (CV = 8%). This might be the consequence of tracking different lineages, as the number of tracked cells increases with time and variability is lower among cells of the same lineage (46). Overall, these results also confirmed the homogeneous behavior, and that the system, as expected, is reversible *in vivo*.

### Molecular characterization of RNA hybridization networks

**Figure 6.**
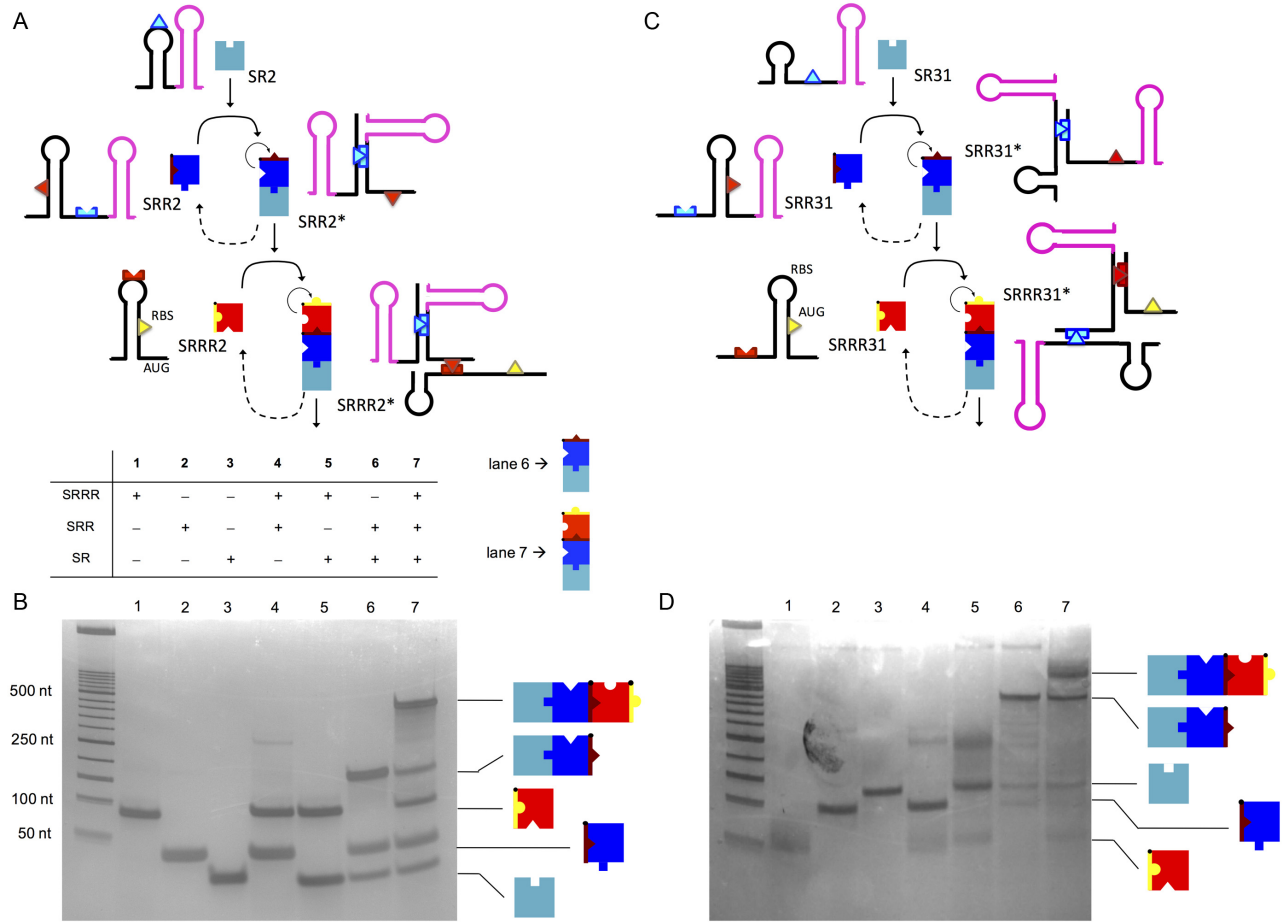
Molecular characterization of designer RNA hybridization networks *in vitro*. (**A, C**) Structures of the species implementing the systems trigR2 and trigR31. The toehold for the interaction between the two sRNAs is shown in light blue. The toehold for the interaction between the heterodimer (sRNA complex) and the 5′ UTR is shown in red. In the 5′ UTR SRRR2 the RBS works as the downstream control element, while in SRRR31 the start codon AUG does (both shown in yellow). The transcription terminator T500 was used in SRR2 and SRR31, while the terminator TrrnC was used in SR2 and SR31. (**B, D**) Electrophoretic analysis showing the hierarchical interaction between sRNAs. The formation of the heterodimer and heterotrimer is shown in lanes 6 and 7, respectively.

To gain mechanistic insight into the hybridizations that define the networks, we characterized the different RNA-RNA interactions by native polyacrylamide gel electrophoresis (PAGE) (38). We chose to analyze the systems trigR2, trigR31 and trigR11, as they represent three different design types. The complementary DNAs corresponding to the RNA species were first transcribed *in vitro* (for the sRNA species without transcription terminators), and then purified and quantified. We mixed the three individual species (SRRR, SRR and SR), and all combinations of two of these species. These mixtures, along with the individual RNAs as controls, were loaded on polyacrylamide gels and separated electrophoretically. The same amount of each RNA per lane was used. For systems trigR2 (Figure 6A; see also Supplementary Figure S12A) and trigR31 (Figure 6C; see also Supplementary Figure S12B), native PAGE analyses revealed the intermolecular interactions between SR and SRR, and between the resulting sRNA complex (SRR*) and SRRR (Figure 6B and D, lanes 6 and 7). Additionally, they revealed a marginal intermolecular interaction between SRR (in the OFF state) and SRRR in trigR2 and trigR31 (Figure 6B and D, lane 4), and also between SR and SRRR in trigR31 (Figure 6D, lane 5). These undesired interactions could be explained, at least in part, by the corresponding free energies of hybridization, which may indeed favor the formation of those complexes (Supplementary Table S3). The electrophoretic analyses also confirmed the intermolecular interactions between SR and SRR, and between SRR* and SRRR for system trigR11 (Supplementary Figure S13).

We also quantified the different species in the electrophoresis gels (39) by considering band intensity proportional to mass. When SR and SRR reacted, we obtained a global mass fraction (mass of SRR* out of the total mass) of 42% in the case of system trigR2, 62% in the case of trigR31, and 21% in the case of trigR11. We also calculated an apparent dissociation constant (by translating the mass fractions into molar fractions; see details in Supplementary Information) for the interaction between SR and SRR of 65 μM for trigR2, 31 μM for trigR31, and 247 μM for trigR11. In addition, when SR, SRR and SRRR reacted, we obtained a global mass fraction (SRRR* over total) of 29% in the case of system trigR2, 28% in the case of trigR31, and 19% in the case of trigR11. We also calculated an apparent dissociation constant for the interaction between SRR* and SRRR of 33 μM for trigR2, 110 μM for trigR31, and 55 μM for trigR11. Taken together, these results show that synthetic RNAs need to be highly expressed to ensure hybridization (47).

### Energetic and structural predictions compared to experimental data

**Figure 7.**
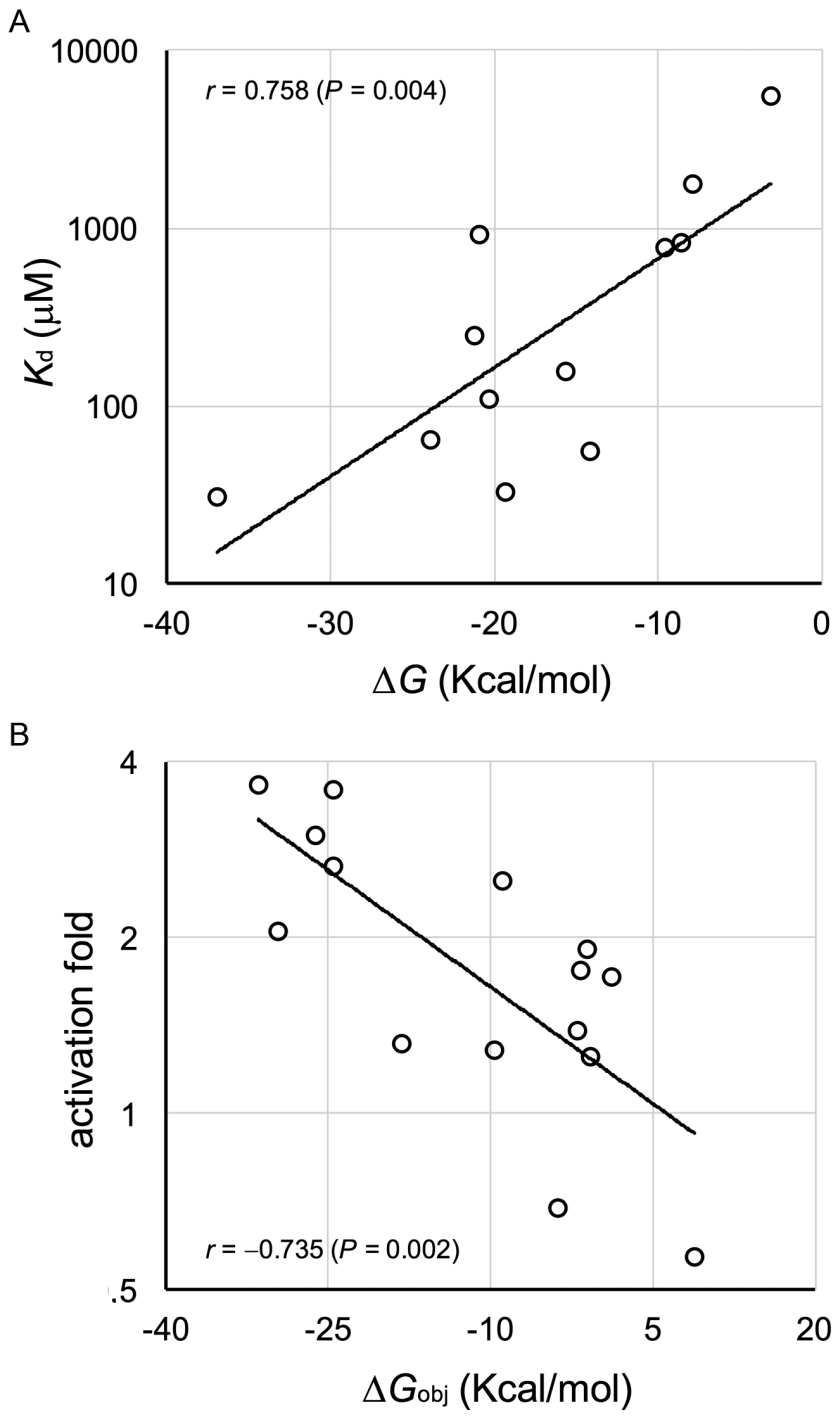
Predicted free energies versus activity of designer RNA hybridization networks. (**A**) Linear correlation between the apparent dissociation constant (in log scale) and the free energy of hybridization. The data shown are for systems trigR2, trigR11 and trigR31, considering the potential interactions between SR and SRR, SR and SRRR, SRR and SRRR, and SRR* and SRRR. Two-tailed Student *t*-based correlation test, *P* < 0.05. (**B**) Linear correlation between the activation fold (in log scale, relative to the uninduced state) and the objective energy of hybridization. The data shown are for all systems, considering the fluorescence increase due to aTc, IPTG and finally aTc and IPTG. Two-tailed Student *t*-based correlation test, *P* < 0.05.

To validate the thermodynamic model, we balanced the computational and experimental results. Supplementary Table S3 shows the free energies that characterize the systems, as predicted by Vienna RNA (26) (see in Supplementary Information how to develop the general objective function expounded in Figure 1B). Subsequently, we quantified, according to our native PAGE analyses (Figure 6 and Supplementary Figure S13), the apparent dissociation constant for each potential interaction, i.e. between SR and SRR (lane 6 in Figure 6B), SR and SRRR (lane 5), SRR and SRRR (lane 4), and SRR* and SRRR (lane 7). We obtained twelve different values for systems trigR2, trigR11 and trigR31, which we compared with the corresponding free energies of hybridization. In this case, the free energy of activation does not matter, because the RNAs were first denatured at 95°C and then cooled to room temperature (38). We found a significant correlation between the experimental constants (in log scale) and the predicted energies (Pearson correlation, *r* = 0.758, *P* = 0.004; Figure 7A), suggesting that the interactions among RNAs are well captured by the model.

In addition, we quantified the activation fold for each induced state (i.e., aTc, IPTG or both) relative to the uninduced state, according to our fluorescence data (Figure 3B). The expression level depends on the degree of de-repression of the 5′ UTR of the mRNA. For each induced state, we defined an objective free energy accounting for that de-repression, which we assessed with the different fold values. In the case of induction with just aTc (or IPTG), it was the sum of the free energies of hybridization and activation between SRR (or SR) and SRRR, as well as the free energy required to de-repress the 5′ UTR in the resulting complex (25). In the case of both inducers, it was the sum of the free energies of hybridization and activation between SRR* and SRRR (having neglected the potential effect of SR and SRR), as well as the free energy required to de-repress the 5′ UTR in SRRR*. Again, we found a significant correlation between the experimental activation folds (in log scale) and the predicted energies (Pearson correlation, *r* = –0.735, *P* = 0.002; Figure 7B), suggesting that our objective function is a well predictor of riboregulatory activity.

Finally, the higher basal expression in the case of trigR31 and trigR32 (i.e., activation with either aTc or IPTG alone) could be explained, at least in part, by the more negative free energies of hybridization between SR or SRR and SRRR. These systems also present larger toehold sequences. However, the contribution of the free energy of toehold hybridization to the reaction kinetics becomes saturated (48), which is in agreement with the similar dynamic range displayed by all engineered systems.

### Extension of the engineered RNA hybridization networks

Larger networks could be engineered provided they do not impose a serious cost for the host cell (here, *E. coli*) (13). The cost can be produced either because the networks consume excessive resources for expression, or because the sRNAs interfere with endogenous mRNAs. Supplementary Table S4 shows the cell growth rates upon expression of our engineered RNA systems in each induction condition, revealing a moderate system-dependent cost. In particular, trigR11 is the costliest system, reducing growth in at most 35%, while trigR1 is the less costly system (the one with the lowest sfGFP expression), with no apparent growth reduction. These data suggest that heterologous protein expression is more determinant of growth reduction than heterologous sRNA expression. Supplementary Table S5 shows potential off-target effects, despite our sequences are fully synthetic, but with no apparent consequence on cell physiology.

For illustrative purposes, we reshaped the network architecture of system trigR31 by incorporating a new RNA species (Supplementary Figure S14) (49). In addition, Supplementary Figure S15 exemplifies, from a theoretical point of view, the ability of RNA hybridization networks to build a computing machine (a simplified case of a Turing machine (50); sequences provided in Supplementary Table S6, based on trigR31 and trigR32; see also Supplementary Figure S16). These extensions are however limited by the dependence on the genetic background of the performance of systems trigR31 and trigR32 (see details in Supplementary Information).

## DISCUSSION

Here, we conceived a general framework for the computational design of RNA hybridization networks to function in living cells (Figure 1). This allows the design of structured RNA molecules with multiple interaction domains, whose activities are conditional to the binding with other molecules, thus resulting in a network of RNA hybridizations. These RNA molecules are hence elements offering novel possibilities for engineering functional, synthetic gene circuits (Supplementary Figure S14), and they add to an increasing toolbox of regulatory RNAs to control gene expression in *trans* and in a combinatorial manner (6,8). We exemplified this by designing different RNA hybridization chain reactions. Designer systems were verified for activity by characterizing the different dynamic ranges with a reporter protein at the population and single-cell levels (Figures 3 and 5), as well as by capturing all possible molecular interactions with native PAGE (Figure 6 and Supplementary Figure S13).

The computational design was possible as nucleic acids are molecules with much higher interaction programmability than proteins (5–8). A thermodynamic model allowed assessing the performance of the different RNA sequences. This way, the sequences implementing the resulting networks (Supplementary Figure S6, Table S1), defined by a set of desired on-target complexes and a set of undesired off-target complexes, satisfy all energetic and structural objectives (Supplementary Figures S1 and S5, Table S3). Here, we used Vienna RNA (26), but other RNA calculators (28,29) could also have been used. Moreover, some of these sequences were designed *de novo* by following a heuristic optimization algorithm (Monte Carlo simulated annealing; Supplementary Figure S2) (5), but other sequences were designed rationally (Supplementary Figure S6f). The *de novo* sequence design could also have been approached by dynamic programming with NUPACK (28,51), as previously done (6).

Nevertheless, the moderate activation fold of our RNA devices might not be adequate for some applications, even though cellular behavior can be reprogrammed (e.g. apoptosis control in eukaryotic cells) with small dynamic ranges (52). Moreover, the correct action of the RNAs might be, in certain cases, modulated by endogenous factors not considered in the design, leading to functional failures of the networks or excessive impact on the physiology of the cell.

Finally, RNA hybridization networks might be very useful to perform bio-logical computations in living cells, due to their ability for storing and retrieving information (e.g. Supplementary Figure S15 shows such ability with the prototype of a computing machine). Future work should be focused on refining and validating experimentally this type of molecular machines and on their exploitation in applied scenarios (53). In addition, RNA hybridization networks might be adapted to organisms other than *E. coli* (including eukaryotic hosts), as they are mainly based on physical interactions. This should be accomplished by only re-designing the interface with the output-protein expression machinery. Certainly, as our ability to design multifaceted RNAs increases, more complex bio-logical computing systems are expected to be developed.

## Supplementary Material

Supplementary Data
