# Supplementary material for: Model-based design of RNA hybridization networks implemented in living cells
Source: Nucleic Acids Res. 2017 Aug 1;45(16):9797–808. doi: 10.1093/nar/gkx698 (PMC5766206; doi:10.1093/nar/gkx698)
Supplement: Supplementary Data [file gkx698_supp.pdf]

Supplementary Information

Model-based design of RNA hybridization networks implemented in living cells

Guillermo Rodrigo, Satya Prakash, Shensi Shen, Eszter Majer, José-Antonio Daròs, and Alfonso Jaramillo

It contains Supplementary Materials and Methods, Additional Texts, 16 Supplementary Figures, and 6 Supplementary Tables.

Supplementary Materials and Methods

Objectives for RNA sequence design

Our approach consisted, first, in developing an empirical thermodynamic model that allowed the computational sequence design [1, 2] and, second, in implementing genetically the designed systems to then characterize the intended behavior. To assess the performance of the RNA molecules, an objective function was calculated with a nucleotide-level energy model considering all conformational states of the system's species (SR, SRR, SRRR, all possible heterodimers, and the heterotrimer), following a combined strategy of positive and negative design. On the one hand, as positive objectives (to be minimized), we considered the free energies of activation and hybridization corresponding to the interactions between the two sRNAs and between the resulting sRNA complex and the 5' UTR. We also considered the interaction between the 5' UTR in complex with the sRNAs and the ribosome. On the other hand, as negative objectives (to be maximized), we took the free energies of activation and hybridization corresponding to the interactions between each sRNA and the 5' UTR. Also, we considered the interaction between the 5' UTR and the ribosome (see Fig. S1). This way, to design our five systems, we combined the *de novo* sequence design, by developing an iterative process of random mutations and selection according to the energy-based objective function (Fig. S2), with the rational sequence design.

Energetic and structural calculations

We used the Vienna RNA package [3] for energy and structure calculation. The calculation of the free energies of full hybridization (ΔG_1 and ΔG_2 for desired interactions, $\Delta \hat{G}_1$ and $\Delta \hat{G}_2$ for undesired ones) was done using the routine cofold. This also gives the final intermolecular structure. To calculate ΔG_2, the free energy of

hybridization between three species, we created a new sequence by simply juxtaposing the sequences of SR and SRR.

The free energies of activation cannot be directly calculated. However, we can write that they (e.g., $\Delta G_1^{\#}$) are related to the free energies of toehold hybridization given an entropic constant (C, i.e., $\Delta G_1^{\#} = C + \Delta G_1^{\text{toehold}}$). This way, the more negative $\Delta G_1^{\text{toehold}}$ is, the closer to 0 $\Delta G_1^{\#}$ is. Thus, the calculation of the free energies of toehold hybridization ($\Delta G_1^{\text{toehold}}$ and $\Delta G_2^{\text{toehold}}$ for desired interactions, $\Delta \hat{G}_1^{\text{toehold}}$ and $\Delta \hat{G}_2^{\text{toehold}}$ for undesired ones) was done again using the routine cofold by only considering the toehold sequences.

Finally, the calculations of the free energies that mediate the interaction between the 5' UTR (either alone or in complex with the sRNAs) and the ribosome (ΔG_3, $\Delta G_3^{\#}$, $\Delta \hat{G}_3$ and $\Delta \hat{G}_3^{\#}$) were approximated for simplicity. We considered that $\Delta G_3 + \Delta G_3^{\#}$ is related to the free energy of the *cis*-repression in SRRR*, and that $\Delta \hat{G}_3 + \Delta \hat{G}_3^{\#}$ is related to the free energy of the *cis*-repression in SRRR. This way, the stronger the *cis*-repression is, the lower translation rate is. By introducing the terms $\Delta G_{SRRR}^{\text{struct}}$ and $\Delta G_{SRRR*}^{\text{struct}}$, as done in ref. [2], we can write $\Delta G_3 + \Delta G_3^{\#} = C' + \Delta G_{SRRR*}^{\text{struct}}$ and $\Delta \hat{G}_3 + \Delta \hat{G}_3^{\#} = C'' - \Delta G_{SRRR}^{\text{struct}}$. These terms were calculated as the Hamming distance between the actual and ideal secondary structures (here, RBS paired in case of SRRR, or unpaired in case of SRRR*) and then considering an average value of 1.2 Kcal/mol per base-pair discrepancy. Note that the terms $\Delta G_{SRRR}^{\text{struct}}$ and $\Delta G_{SRRR*}^{\text{struct}}$ are positive. In addition, note that the free energies characterizing the interaction with the ribosome could also be calculated following the function proposed in ref. [4]. Further work could incorporate this to improve the accuracy of the objective function.

The resulting objective free energy to be minimized is $\sum u_{ij}$ ($\Delta G_{ij} + \Delta G_{ij}^{\#}$) =

$\Delta G_1 + \Delta G_1^{\text{toehold}} + \Delta G_2 + \Delta G_2^{\text{toehold}} - \Delta\hat{G}_1 - \Delta\hat{G}_1^{\text{toehold}} - \Delta\hat{G}_2 - \Delta\hat{G}_2^{\text{toehold}} + \Delta G_{SRRR}^{\text{struct}}$ $+ \Delta G_{SRRR*}^{\text{struct}}$. See Figs. S1 and S5 for illustrative purposes.

Notes on RNA sequence design

In case of systems trigR31 (or trigR32), element SR31 (or SR32) is directly the sRNA of system 1 (or 7) from ref. [5] [taken from plasmid pAG_TS2_AT01 (or pAG_TS2_AT07)], but using a bacterial terminator. Element SRR31 (or SRR32) is a modification of the cognate 5' UTR [plasmid pAG_TS2_KS01 (or pAG_TS2_KS07)], without linker and carrying a mutation to disrupt the RBS (GG $\rightarrow$ CC), also with a bacterial terminator. Then, a 5' UTR responsive to SRR31* (or SRR32*) was designed keeping those sRNA sequences fixed (see Table S1).

In case of system trigR11, the toehold is not hidden within the corresponding intramolecular structure (of SRR11), but it still remains inactive. This is because the hybridization free energy is not sufficient to ensure irreversible interaction (with SRRR11), and an additional species (SR11) is required for the reaction. The free energy of hybridization between SRR11* and SRRR11 is then sufficient to form the triple intermolecular folding state with a three-way junction. When constructing SRR11 and SR11, we found that both sRNAs had an active toehold that allowed them to interact. The heterodimer SRR11* has another active toehold that nucleates its binding to SRRR11 by forming a heterotrimer with the three-way junction (see Table S1 and Fig. S7).

To design the RNA elements that implement the molecular machine shown in Fig. S15, we relied on systems trigR31 and trigR32 and applied computational design to redesign the sequences. The element that work as record tape (SRtape) was derived from SR31 by adding a hairpin with the second toehold hidden. We also redesigned the

elements SRR31 and SRR32 (called SRR31bis and SRR32bis) to, on the one hand, interact with the record tape and, on the other hand, still interact with the cognate 5' UTRs (see Table S6). The elements that control the expression of the two reporter proteins in the 5' UTR are directly the elements SRRR31 and SRRR32. This way, SRtape interacts first with SRR31bis, and then with SRR32bis.

Additional plasmid construction

For systems trigR11 and trigR2, we also constructed a variant with a non-tagged sfGFP. In the construction of the control circuit where the two sRNAs are fused transcriptionally, the promoter P_{LtetO1} was used. In the construction of the circuit with two regulatory branches (Fig. S14), the element SRRb31:sfGFP was expressed from promoter J23119 (see Table S2).

Preparation for *in vitro* RNA-RNA interaction

We first constructed the cDNAs of the different RNA species of the designed system to then perform the *in vitro* transcription. We analyzed the systems trigR2, trigR11 and trigR31. We considered the sRNAs without transcription terminators and the 5' UTR until the start codon. Amplification by PCR (30 cycles, extension 0.5 min), using Phusion DNA polymerase (Thermo Scientific), was done over the template plasmid (ptrigR2, ptrigR11 or ptrigR31). The PCR products were cloned into the plasmid pUC18, where the restriction site Eco31I was previously removed. The resulting plasmids with inserts were selected by DNA cleavage with appropriate restriction enzymes. Sequences were also verified by sequencing.

In case of trigR31, element SRRR31 was not *in vitro* transcribed (presumably due to strong secondary structure), so it was digested with Esp3I to get a shorter RNA but still able to interact with the other RNAs.

For the reaction of RNA-RNA interaction, we used approximately the same amount of RNA for each of the transcripts (20 ng for systems trigR2 and trigR11, and 60 ng for system trigR31).

Apparent dissociation constant estimation

ImageJ was used to quantify the intensities of the bands [6]. We mainly focused on two lanes: the lane having the two sRNAs (species SR and SRR; to quantify the dimeric interaction), and the lane having the two sRNAs and the 5' UTR (species SR, SRR and SRRR; to quantify the trimeric interaction). With these intensities, we calculated the different mass fractions. Moreover, by knowing the RNA sequences, we translated band intensities (proportional to mass) into molar concentrations. Note that sequences could be longer in the 5' end (including GG when needed for T7 RNA polymerase) or shorter in the 3' end (excluding transcription terminators). The apparent dissociation constants were estimated by dividing the resulting molar concentrations of the reactants with respect to the products, i.e., [SR]·[SRR] / [SRR*] in case of sRNA-sRNA interaction, and [SRR*]·[SRRR] / [SRRR*] in case of (sRNA:sRNA)-5' UTR interaction.

Living cells and energy gains for fluorescence quantification

For characterization in the Infinite F500 multi-well fluorometer (TECAN), plasmids carrying all the systems (trigR31, trigR32, trigR11, trigR1 and trigR2) were transformed into DH5α-Z1 cells. The systems trigR31 and trigR32 were also

characterized in MG1655-Z1 cells, because their activity in DH5α-Z1 cells was marginal.

For systems trigR31, trigR11 and trigR1 the gain of the fluorometer was set to 35, for system trigR2 to 25 (due to strong translation rate), and for system trigR32 to 45 (due to weak translation rate). Fluorescence values were then rescaled according to the scale of gain 35.

Additional Texts

Rationale about the interaction with the ribosome

An efficient interaction between the 5' UTR of an mRNA (SRRR element) and the ribosome requires that both the RBS sequence (Shine-Dalgarno, SD) and the start codon (AUG) are within an unpaired structural context [4], i.e., not only the SD and AUG nucleotides but also the surrounding nucleotides. Thus, for an efficient *cis*-repression of translation initiation, both the RBS sequence and the start codon have to be within a paired structural context. However, this condition can be relaxed, as shown experimentally. Only the *cis*-repression of the RBS or the start codon is required to construct riboregulatory systems [1, 5]. Following these design principles, we here constructed different SRRR elements. The SRRR elements in the case of trigR1, trigR2, and trigR11 were designed by specifying the objectives of RBS occlusion in the OFF state and release in the ON state. By contrast, the SRRR elements in the case of trigR31 and trigR32 were designed by specifying the objectives of AUG occlusion and release. The resulting structure of the SRRR element in the case of trigR32 also revealed a *cis*-repression of the RBS, although it is not the system with lower expression.

Effect of the genetic background on the performance of the regulatory systems

Systems trigR31 and trigR32 were designed based on two riboregulators previously engineered and characterized [5] (see above *Notes on RNA sequence design*). In our characterizations at the population level, these systems exhibited, unexpectedly, a marginal activity in DH5α-Z1 cells. We then performed new characterizations in MG1655-Z1 cells, obtaining better results in terms of activity. Results shown in main Fig. 3 for these systems correspond to expressions in MG1655-Z1 cells. The other systems (trigR11, trigR1 and trigR2) displayed similar activities in both cell types.

Equilibrium of RNA-RNA interactions

We can assume that the underlying RNA-RNA interactions of our systems are in thermodynamic equilibrium [7]. This way, we can explain the increase in protein expression as a function of the concentrations of the RNAs. We can state that protein expression depends on the concentration of complex SRRR*. The formation of this complex in turn depends on the concentrations of SR, SRR and SRRR, as well as on the equilibrium constants of the two RNA-RNA interactions in chain.

On the one hand, the promoters P_{LlacO1} and P_{LtetO1} produce, at most, an expression level of the two sRNAs of 1-10 μM (with IPTG and aTc in the Z1 background) [8]. Moreover, the promoter J23119 may produce an expression level of the mRNA of 1 μM. Note that these expression values are estimated for a high-copy number plasmid.

On the other hand, according to our *in vitro* RNA-RNA interaction results, the effective dissociation constants are in the range of 30-300 μM. In particular, for system trigR2, we obtained K_1 = 65 μM for the SR-SRR interaction (forming the complex SRR*), and K_2 = 33 μM for the SRR*-SRRR interaction (forming the complex SRRR*). These constants depend on the free energies of hybridization and activation, as previously shown [9]. In particular, we can write $K_1 \sim \exp[\beta\,(\Delta G_1 + \Delta G_1^{\#})]$ and $K_2 \sim \exp[\beta\,(\Delta G_2 + \Delta G_2^{\#})]$, where and β is a fitting constant (see also main Fig. 7). That is, lower the free energy of hybridization (i.e., more negative), lower the dissociation constant (i.e., higher the equilibrium constant, higher affinity). And higher the free energy of activation (i.e., more positive), higher the dissociation constant (i.e., lower the equilibrium constant, lower affinity).

Because the values of the effective dissociation constants are much higher than the expected concentrations of the sRNA molecules within the cell, we can state that the system is in a linear regime. Hence, following previous calculations [9], we can write [SRR*] = [SR]·[SRR] / K_1, and [SRRR*] = [SRR*]·[SRRR] / K_2. Combining these two equations, we get [SRRR*] = [SR]·[SRR]·[SRRR] / K_1K_2, i.e., we obtain the concentration of the final complex as a product of the concentrations of the initial species. This gives a plane in log scale. Note that the expression of SRRR* could be enhanced either by mutations that modify the free energies or by increasing the concentrations of the sRNAs, both factors reshaping the equilibrium.

A model-based prediction of the formation of the complex SRRR* as a function of the concentration of the species SR and SRR, together with the experimental data of fluorescence by varying the concentrations of IPTG and aTc, is shown in Fig. S9 for system trigR2. The difference between the two surfaces may be attributed to the nonlinearity introduced in the transcription process.

Coupling of an RNA hybridization network with the cellular machinery

In vivo, sRNAs may interact with the cellular machinery to perform their functions. In *E. coli*, sRNAs often interact with the Hfq protein, which acts as a chaperone to stabilize and facilitate the binding to their targets [10]. We did not consider such interactions in our model, so we asked if we would get enhanced functionality by rationally engineering an interaction with Hfq. To this end, we created an additional system based on trigR31, which showed the less-prominent digital behavior (see main Fig. 3). We introduced an Hfq target in SRR (sequence MicF-M7.4 from ref. [11]), with the aim of increasing the interaction between SRR* and SRRR and then shifting the equilibrium towards the formation of SRRR*. But we obtained a similar result (Fig. S10a). It is

possible that fewer Hfq molecules were available in the cell compared to the expected high number of SRR molecules, and that they were hence insufficient to affect the system [10, 12] (note that the SRR was highly expressed from a plasmid, so the relative number of SRR:Hfq molecules would be low). Although more research is certainly needed, the engineering of Hfq interactions seems unlikely to increase performance in highly expressed RNA hybridization networks with optimized free energies.

Creation of a simple riboregulator from an RNA-triggered riboregulator

As we constructed an RNA-triggered riboregulator from a simple riboregulator (system trigR11; Fig. S7), we asked if the converse operation would be possible. To this end, we created a new riboregulator by transcriptionally fusing the species SR and SRR (resulting in the new species SR-SRR) from system trigR31. We then tested the new riboregulator *in vivo*, which showed a similar activation of gene expression than the original system (Fig. S10b).

Note on off-target effects

When expressing heterologous sRNAs *in vivo*, it is important to take into account that these molecules can interact by antisense mechanism with endogenous mRNAs, then producing some undesired effects on the chassis cell. Table S5 shows eventual off-target effects of some of our designed riboregulators using RNApredator [13] (considering the 5' UTRs of all mRNAs in the genome of *E. coli* K-12 MG1655), although the viability of the cell (effects on essential genes) was not compromised when expressing them (our experiments showed that bacterial cells grew normally).

Engineering combinatorial regulation with RNA-triggered riboregulators

To illustrate the ability of designing and implementing combinatorial regulatory circuits with sRNAs in living cells, we engineered a system with two regulatory branches as a proof of concept. For that, we considered our system trigR31 and took from previous work [5] the sequence of the 5' UTR (SRRb) that is responsive to the sRNA SR. We placed this *cis*-repressing element together with a sfGFP under the control of a constitutive promoter (implemented in another plasmid). This way, SR can activate sfGFP by two routes, one with an intermediate sRNA (SRR; Figs. S14a,b). Figure S14c shows the dynamic range (characterized by fluorometry) of the system, probing the interoperability of different layers of sRNAs with the allosteric toehold activation mechanism.

RNA hybridization networks for computation in living cells

To illustrate the potential applications of our engineered systems, we conceived a simplified version of a Turing machine [14] to perform computations with genetically-encoded RNAs inside a living cell, in the line of previous work *in vitro* [15, 16]. Indeed, the exploitation of RNA molecules for storage and retrieval of information *in vivo* allows the modulation of gene expression profiles according to a set of instructions processed by a machine that encodes a set of predefined rules. In our design (Fig. S15a), the machine (called Turing *head*) is implemented by RNA-triggered riboregulators, and it is able to activate predetermined gene expression programs upon reading arbitrary information linearly stored in a heterologous RNA molecule (called *tape* in the context of Turing machines). We conceived the Turing head relying on species SRR and SRRR. These RNA molecules are appropriately disposed to interact with (read) the tape, while the expression levels of different *cis*-repressed genes (A, B, etc.) register the internal state. Moreover, a multi-toehold SR molecule plays the role of the tape (the SRtape

molecule), and only one toehold (the symbol currently read) is active at a time, with an arbitrary number of hidden toeholds. The set of all possible toeholds (symbols) is the alphabet of the machine, which performs arithmetic operations according to a predefined transition state table (Fig. S15b). Upon hybridization with the cognate SRR molecule, the tape moves to the left so that the Turing head is able to read the next symbol (see Fig. S16 for an illustration of this movement for different SRtape molecules). This mechanism can be used in series to progressively read toeholds and activate genes. Of note, the intended machine registers the final state, in the form of gene expression, but does not write on the tape.

To exemplify the implementation of such a Turing machine, we here exploited the systems trigR31 and trigR32 (Fig. S15c), showing that it is possible to achieve complex computations by only relying on RNA hybridization networks. For that, we redesigned the molecules SRR31 and SRR32 (now called SRR31bis and SRR32bis, respectively). The 5’ UTRs of the registry genes are directly SRRR31 and SRRR32. We also designed the new molecule SRtape to contain one toehold active and another inactive (sequences shown in Table S6). In this design, the tape has two toeholds, but it could have a larger number. This way, the Turing head reads the first active toehold through SRR31bis, triggers a regulatory cascade that activates gene A controlled by SRRR31, and the subsequent toehold in SRtape becomes active (state A in Fig. S15c).

It is interesting to make an analogy to the translation machinery [17], where the symbols in the SRtape molecule would play the role of codons and the SRR molecules the role of tRNAs.

The SRtape molecule could be introduced in the cell as DNA through horizontal gene transfer mechanisms, which would allow transferring digital information among cells. This could lead to the development of RNA-based distributed computation

platforms exploiting cell populations [18], as done with the signaling of small molecules affecting transcription factors [19].

The strategy presented here would allow the development of a read-only Turing machine, but it should be possible to use RNA to design a writing system. For that, the DNA sequence coding for the SRtape would be modified. Recombination-based methods [20] or the type IE CRISPR system [21] have been recently used to store information in DNA, illustrating that the approach could be feasible.

The cost of complex RNA-based computations

The use of RNA to implement the computations would reduce the size of the DNA piece required for encoding and would enlarge considerably the alphabet of available symbols. Indeed, a tape made of RNA of 30 instructions could be encoded in place of a single protein of average size. However, the execution of complex RNA-based programs would require the expression of a large number of molecules (sRNAs), which could impact on the cell growth rate. In particular, 10^4-10^5 molecules seem to be required for a network of two nested interactions (according to main Fig. 7a). To overcome the cost of expressing all sRNAs at a time, our RNA hybridization networks could be interfaced with RNA-guided transcriptional control mechanisms [22, 23] to turn off the unsolicited species at a given point.

Natural RNA-triggered riboregulators?

The sequences implementing our systems are fully synthetic, but appropriate bioinformatic approaches [24, 25] might unveil natural examples of RNA-triggered riboregulators. This would constitute a new layer in the host riboregulome.

Supplementary Figures

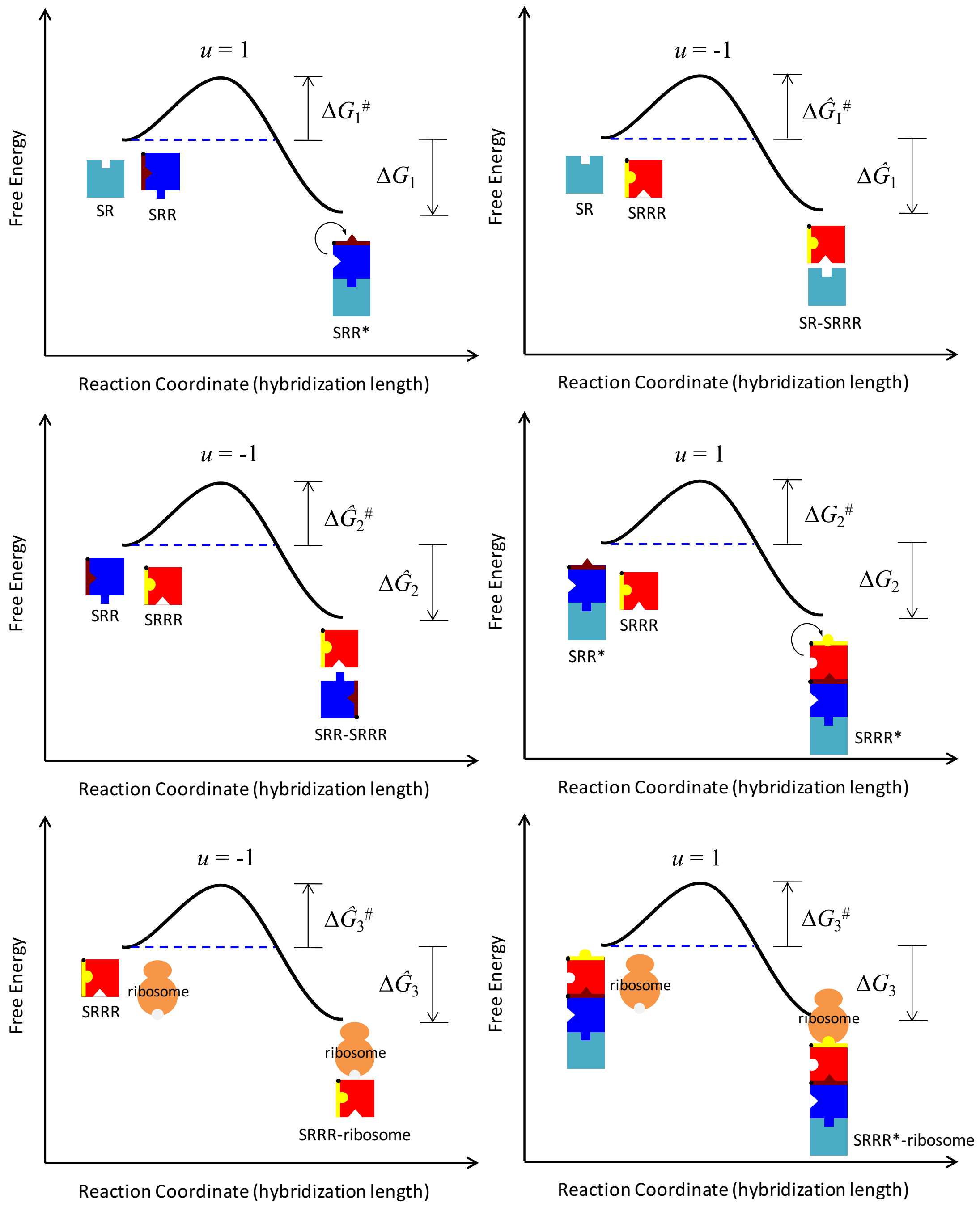

Figure S1: Illustration of all energetic terms used to design an RNA hybridization chain reaction. Here, SRRR is the expression platform (i.e., mRNA). Note that the free energy of hybridization is a negative magnitude, whereas the free energy of activation is a positive magnitude. Moreover, u indicates positive (1, energy minimization) or negative (-1, energy maximization) design. This way, the objective function to be minimized is $\Delta G_1 + \Delta G_1^{\#} - \Delta\hat{G}_1 - \Delta\hat{G}_1^{\#} + \Delta G_2 + \Delta G_2^{\#} - \Delta\hat{G}_2 - \Delta\hat{G}_2^{\#} + \Delta G_3 + \Delta G_3^{\#} - \Delta\hat{G}_3 - \Delta\hat{G}_3^{\#}$.

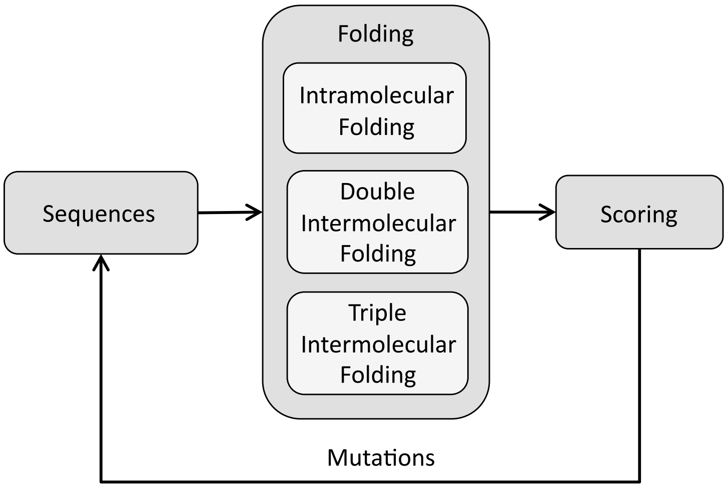

Figure S2: Scheme of the optimization loop, where three RNA sequences (SR, SRR, and SRRR) are iteratively mutated and evaluated according to the objective function. To fold the sequences, we used ViennaRNA [3].

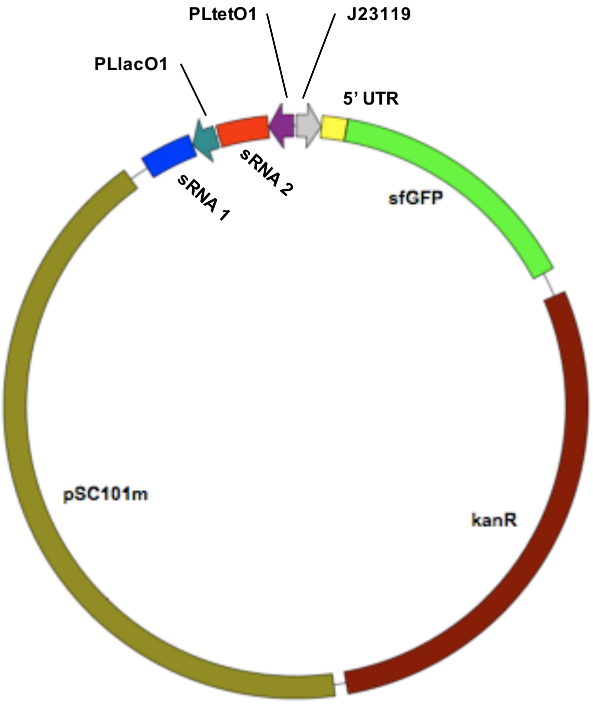

Figure S3: Map of the plasmid used in this work for expressing the designed sRNA systems. The sRNAs 1 and 2 correspond to SR and SRR, respectively, according to our terminology. The 5' UTR is named as SRRR.

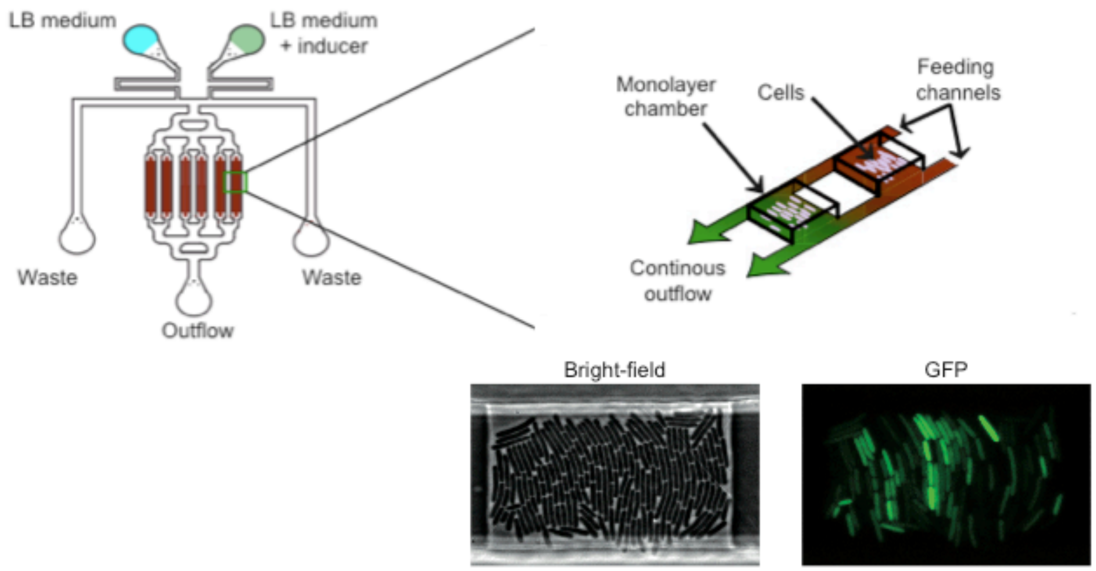

Figure S4: Scheme of the microfluidic device used to monitor GFP expression in single cells (see ref. [26] for a review of this technique). The device can receive two different input media, either LB or LB with inducers. Bacterial cells are loaded into the device and trapped in the microchambers. They are exposed to a continuous flow of media. Cell images from the bright-field channel serve for segmentation and tracking. Images from the fluorescence channel can be quantified.

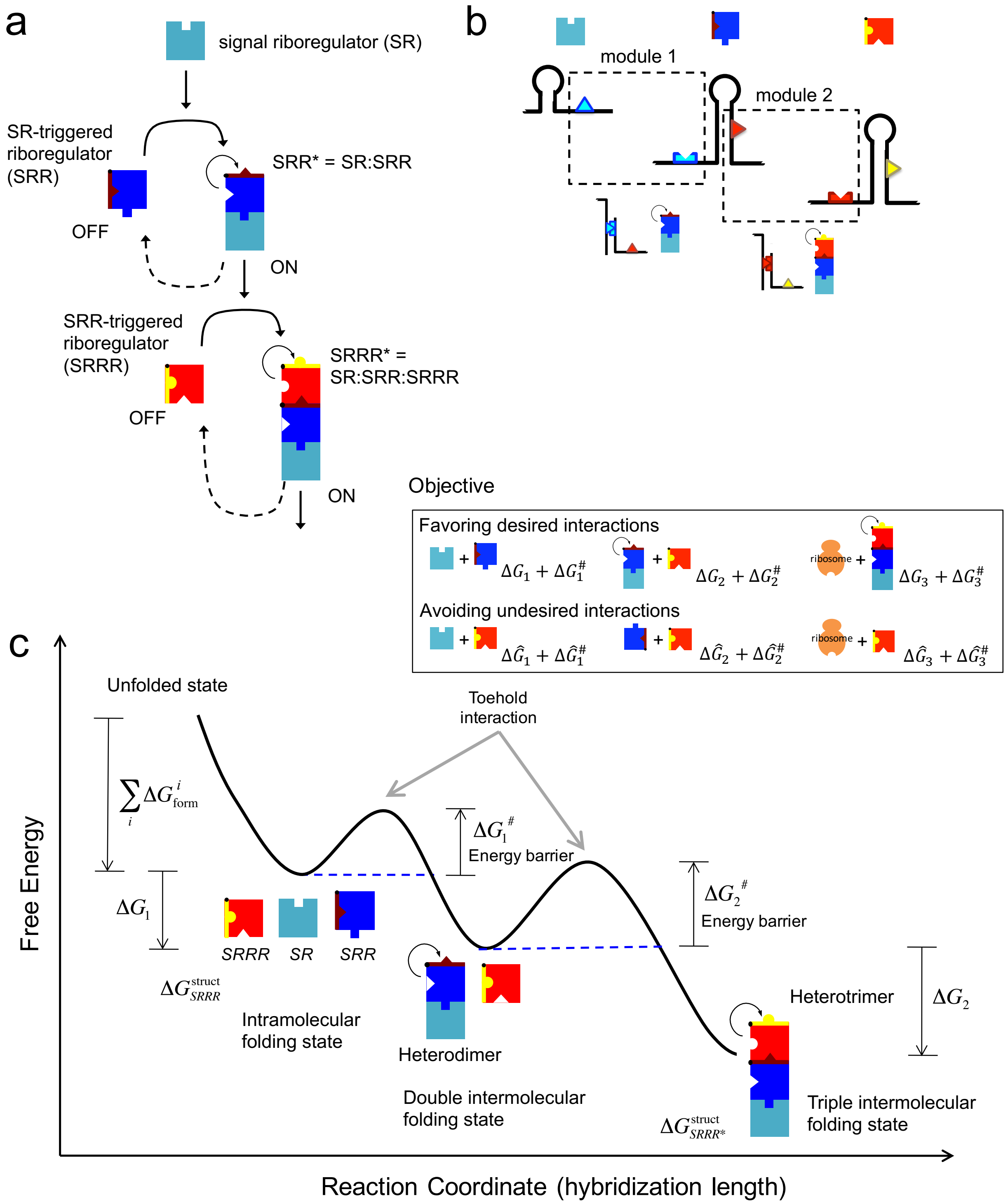

Figure S5: (a) Scheme of riboregulatory cascades implemented with RNA-triggered riboregulators (i.e., riboregulators that allosterically switch from an OFF state to an ON state upon interaction with another riboregulator). (b) Structural implementation of the cascade with allosteric programming of toehold activation. Two different interaction modules are identified. (c) Energy landscape of riboregulatory cascades (here of three molecules, named SR, SRR and SRRR). The energy landscape shows the different conformational states (intra- and intermolecular), together with the free energy terms of the objective function, as a function of a reaction coordinate (number of intermolecular base pairs). In the inset, the objective function is illustrated.

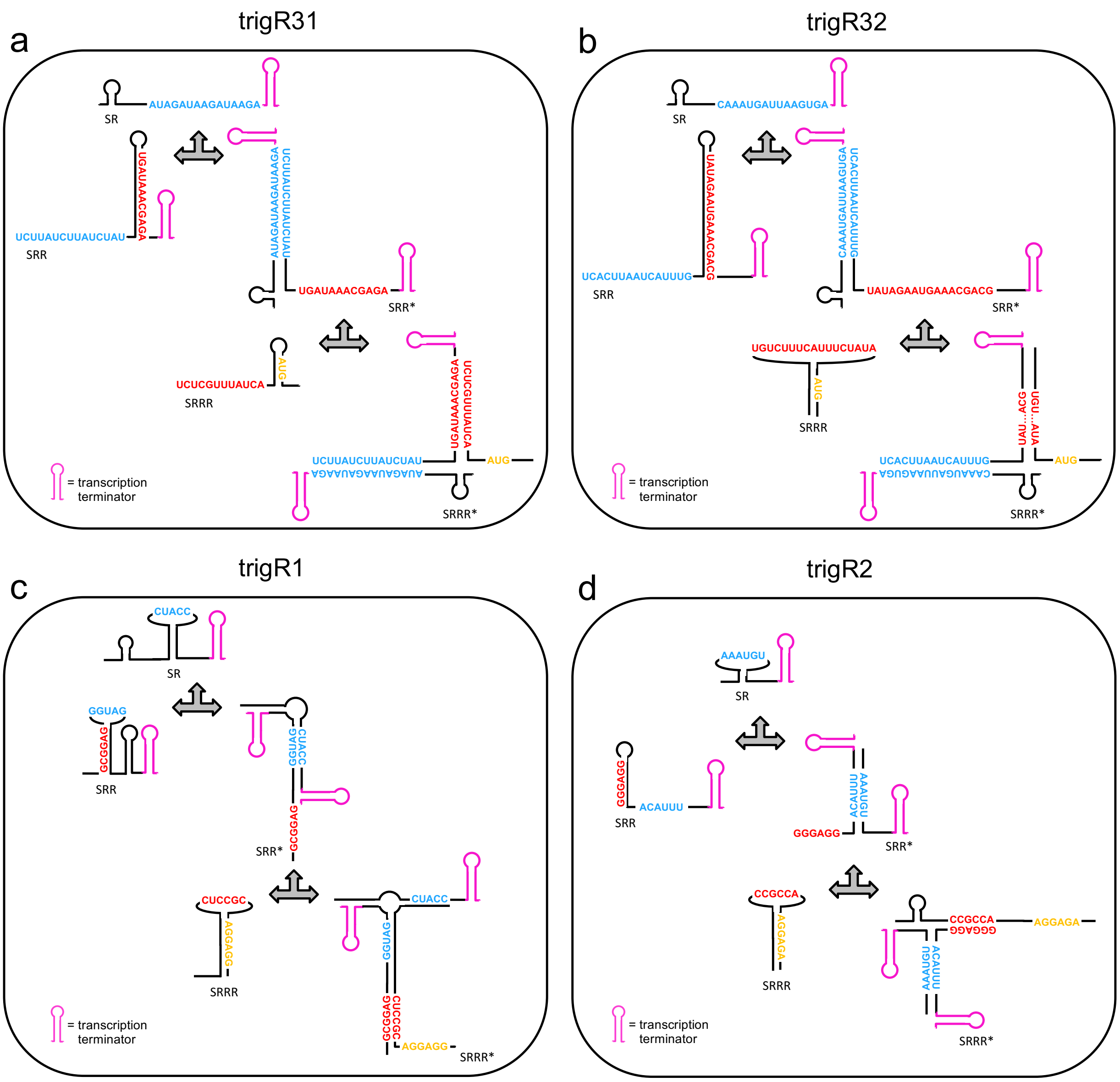

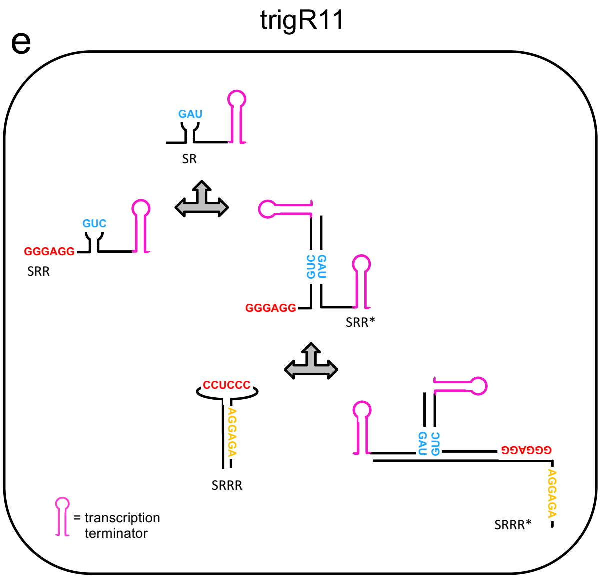

f

Molecule System	SR	SRR	SRRR
trigR31	previously designed	adapted from previous design	*de novo* designed
trigR32	previously designed	adapted from previous design	*de novo* designed
trigR1	*de novo* designed	*de novo* designed	*de novo* designed
trigR2	*de novo* designed	*de novo* designed	*de novo* designed
trigR11	adapted from previous design	adapted from previous design	previously designed

Figure S6: Sequence-structure schematics of designer RNA hybridization chain reactions: (a) trigR31, (b) trigR32, (c) trigR1, (d) trigR2, and (e) trigR11. For each system, the toehold sequence for the interaction between the two sRNAs is shown in blue, and the toehold sequence for the interaction between the heterodimer (sRNA complex) and the 5' UTR is shown in red. See Table S1 to know what transcription terminator (depicted in pink) is used in each sRNA. In the 5' UTR, the RBS or the start codon AUG (shown in yellow) works as the downstream control element. (f) Report about how the different sequences were obtained.

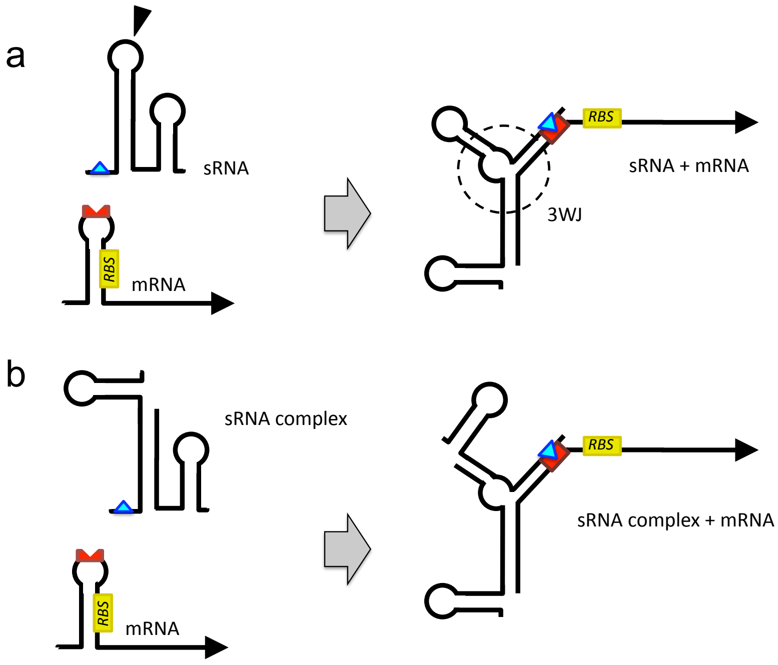

Figure S7: (a) Scheme of the riboregulatory system RAJ11 (one sRNA interacts with the 5' UTR of mRNA) [1]. (b) Scheme of the cooperative riboregulatory system trigR11 (two sRNAs form a complex that interacts with the 5' UTR). This system is based on the previous one by taking advantage of the three-way junction (3WJ) formed to then split the sRNA in two at the wedge (and add a terminator to the first fragment). The sRNAs are illustrated with terminators.

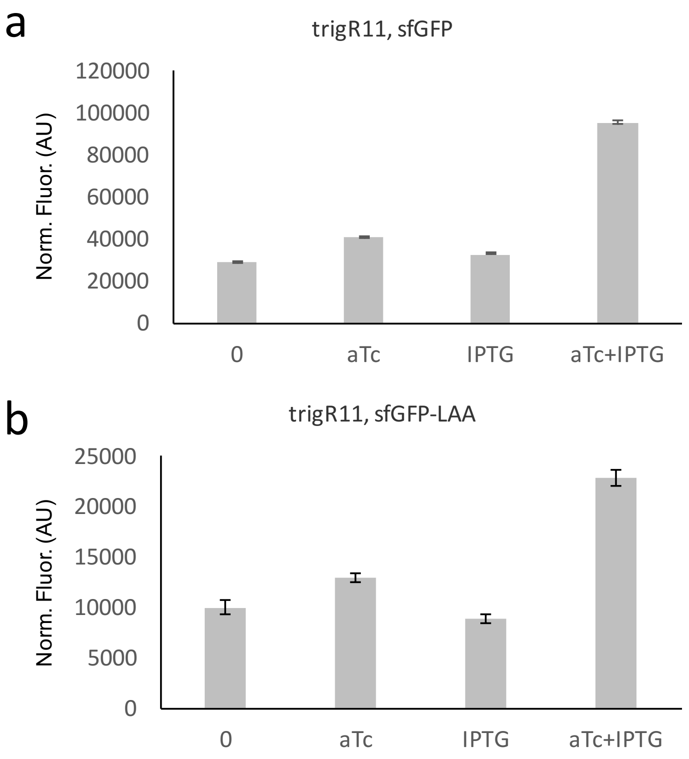

Figure S8: (a) Functional characterization of the designed sRNA system trigR11 with the reporter sfGFP without degradation tag. (b) Comparison against a recharacterization of that system in the same conditions using the tagged sfGFP (LAA). Three replicates. In those systems where the basal expression level is high (e.g., trigR2), a characterization with the non-tagged sfGFP gives non-significant differential expression due to saturation.

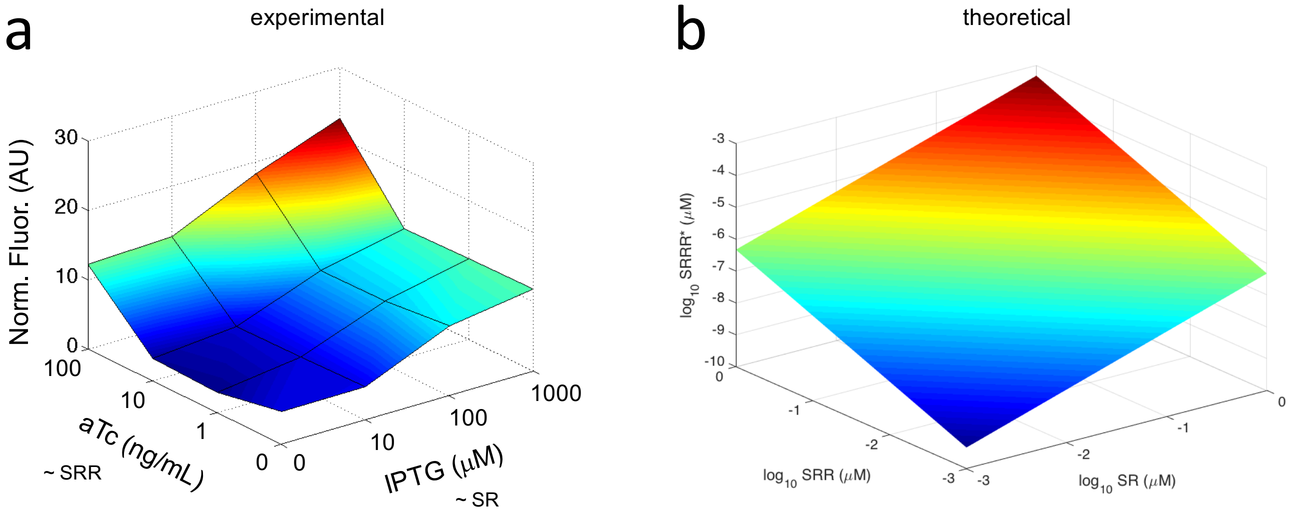

Figure S9: Effect of the concentrations of the sRNA molecules on the expression of the target gene. (a) For system trigR2, fluorescence results are shown for a gradient of IPTG and aTc. IPTG controls the expression of the sRNA SR, whilst aTc the expression of the sRNA SRR. (b) Model-based prediction of the formation of the complex SRRR* as a function of the concentration of the species SR and SRR, given a constant amount of the species SRRR (assumed 1 μM). See above *Equilibrium of RNA-RNA interactions*.

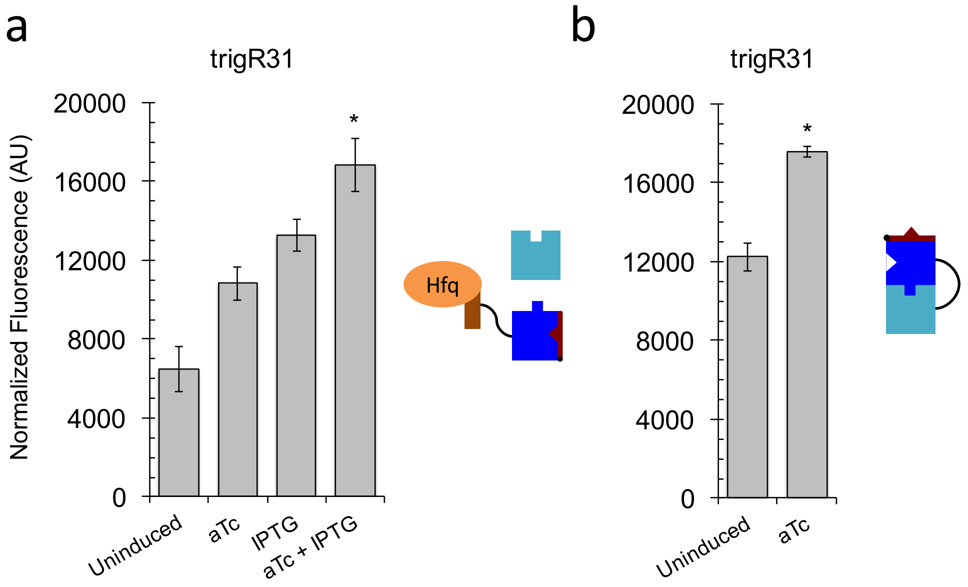

Figure S10: (a) Fluorescence results of two control systems based on trigR31 by introducing an Hfq scaffold in SRR, and by fusing transcriptionally the sRNAs SR and SRR. Three replicates. The differential expression is significant (one-tailed Welch *t*-test, $P < 0.05$; labeled with an asterisk).

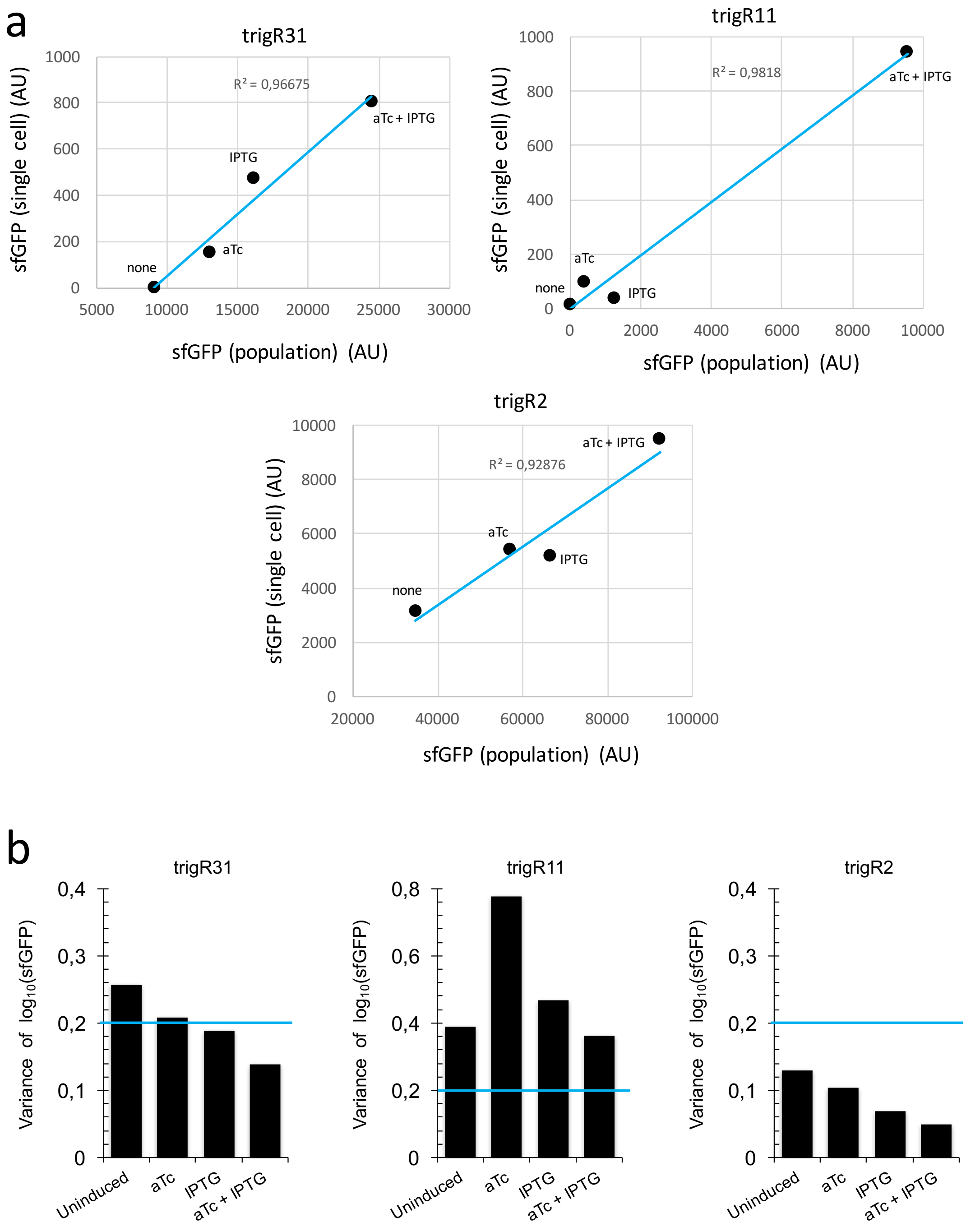

Figure S11: (a) Comparison between the activity of the designed sRNA systems trigR31, trigR11 and trigR2 at the population (by fluorometry, data for one clone) and single-cell levels (by flow cytometry, data for one clone). (b) Variance of sfGFP expression according to the single-cell data of the designed systems. The horizontal blue line corresponds to the variance reported for the system RAJ11 (simple riboregulation) upon induction [1] for a comparative.

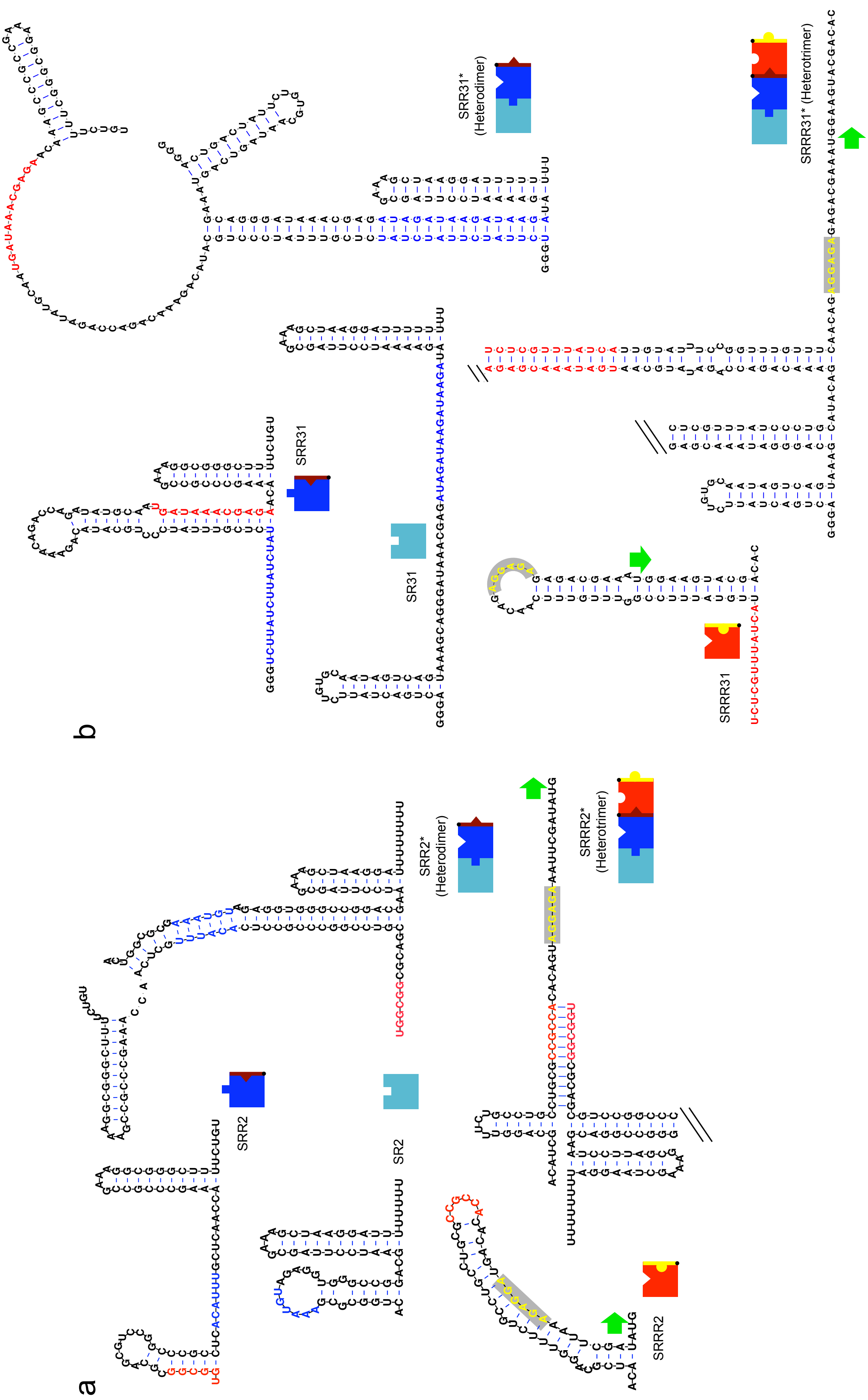
a
b
SRR2
SR2
SRR2
SRR2* (Heterodimer)
SRR2* (Heterotrimer)
SRR31
SR31
SRRR31
SRR31* (Heterodimer)
SRRR31* (Heterotrimer)

Figure S12: Sequences and structures of the species of the designed sRNA systems trigR2 (a) and trigR31 (b). The toehold for the interaction between the two sRNAs is shown in blue. The toehold for the interaction between the heterodimer (sRNA complex) and the 5' UTR is shown in red. In the 5' UTR (SRRR2 or SRRR31), the RBS is shown in yellow and the start codon marked by a green arrow. The transcription terminators T500 and TrrnC were used in SRR2 or SRR31 and in SR2 or SR31, respectively (see Table S1).

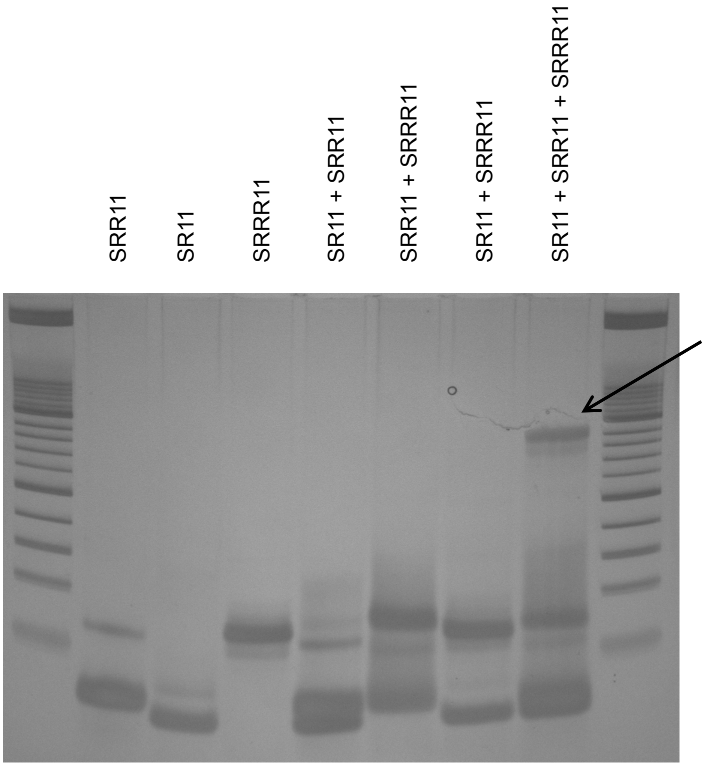

Figure S13: Electrophoretic analysis of system trigR11. The different lanes correspond to all combinations of species. The arrow marks the interaction of the three RNAs.

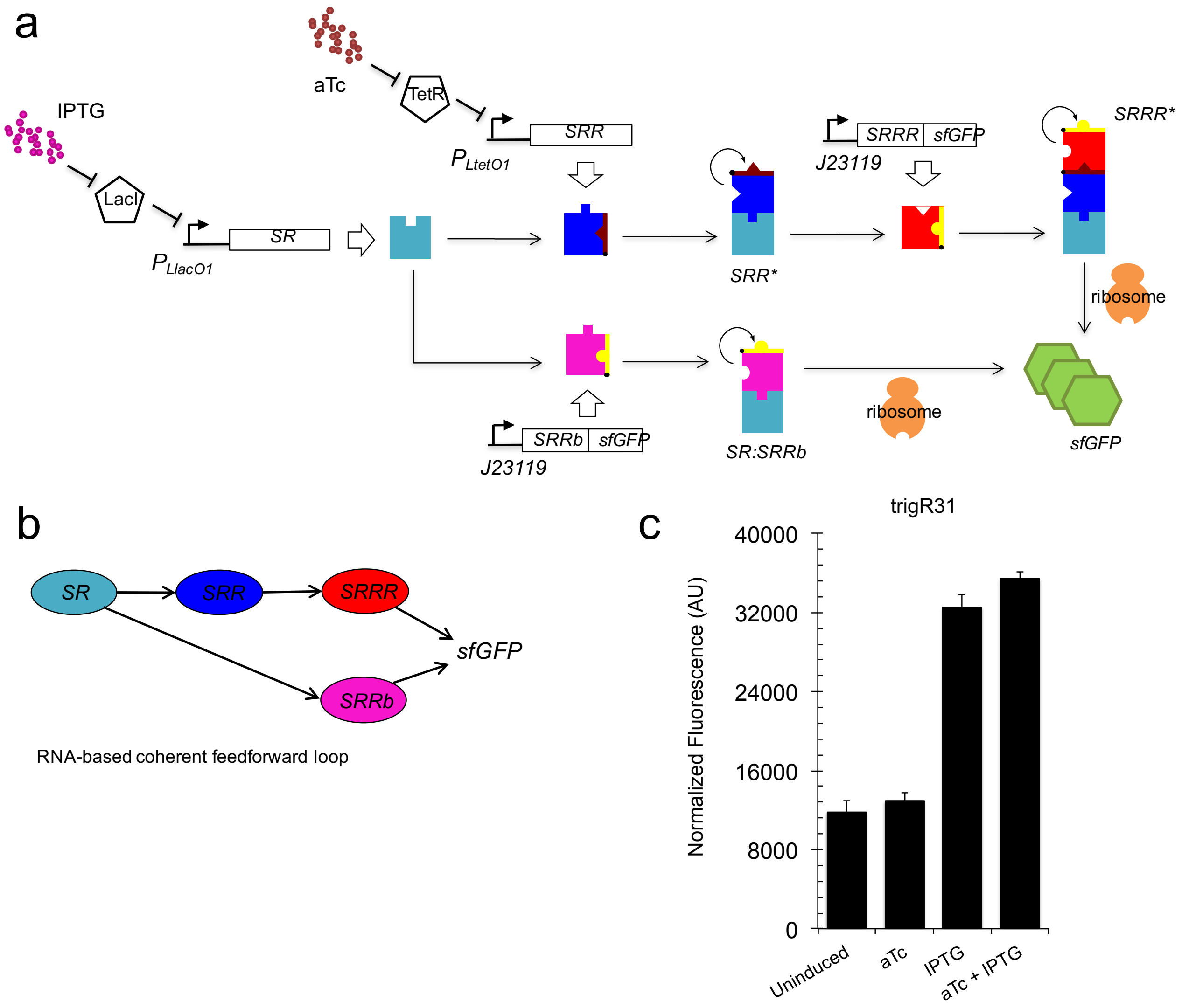

Figure S14. Functional characterization of designer feedforward loop gene circuit with riboregulatory cascades in bacterial cells. (a) Scheme of the engineered sRNA circuit. Promoters P_{LlacO1} and P_{LtetO1} control the expression of the two sRNAs (SR and SRR), which can be tuned with external inducers IPTG and aTc, whereas the two mRNAs (SRRR:sfGFP and SRRb:sfGFP) are constitutively expressed from promoter J23119. SR can directly activate one *cis*-repressed gene (SRRb:sfGFP), and the second *cis*-repressed gene (SRRR:sfGFP) is activated by the complex formed by the two sRNAs upon interaction (SRR*). The reporter gene is a sfGFP. (b) Minimal scheme of the feedforward loop circuit. (c) Fluorescence results (arbitrary units, AU) of the engineered circuit based on system trigR31 for all possible combinations of inducers. Error bars represent standard deviations over three replicates.

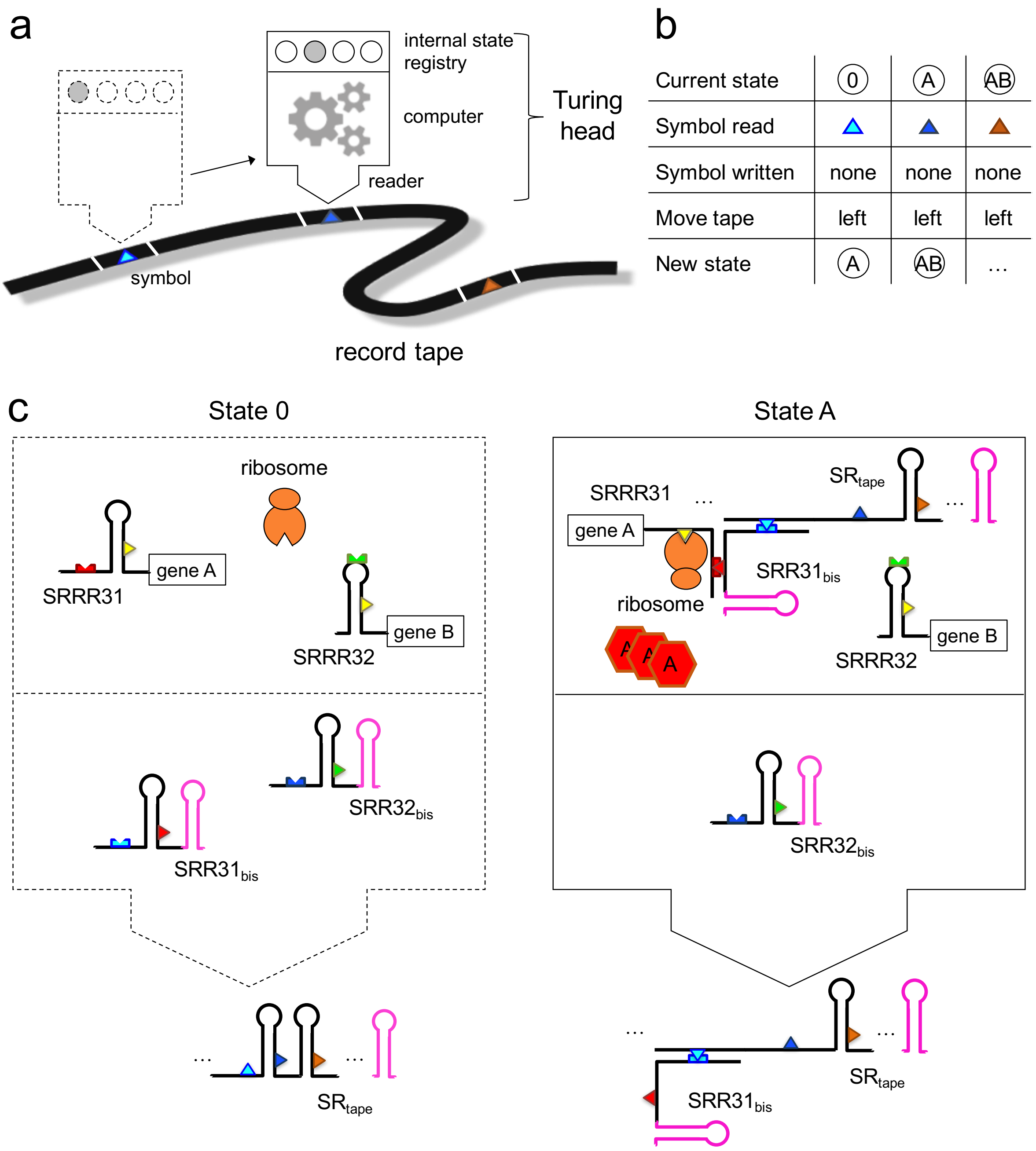

Figure S15. (a) General scheme of a Turing machine, where the head is positioned over the tape to read the symbols and then perform computations. Here, we consider a machine that reads but does not write on the tape. (b) Exemplification of a transition state table of the abstract machine. The instructions are implemented as 5-tuples, which given the current state of the machine and the symbol to be read in the tape dictate the new state of the machine, the symbol to be written instead, and the movement of the tape. (c) Implementation of the machine as an RNA hybridization network. We illustrate the transition from one state to another.

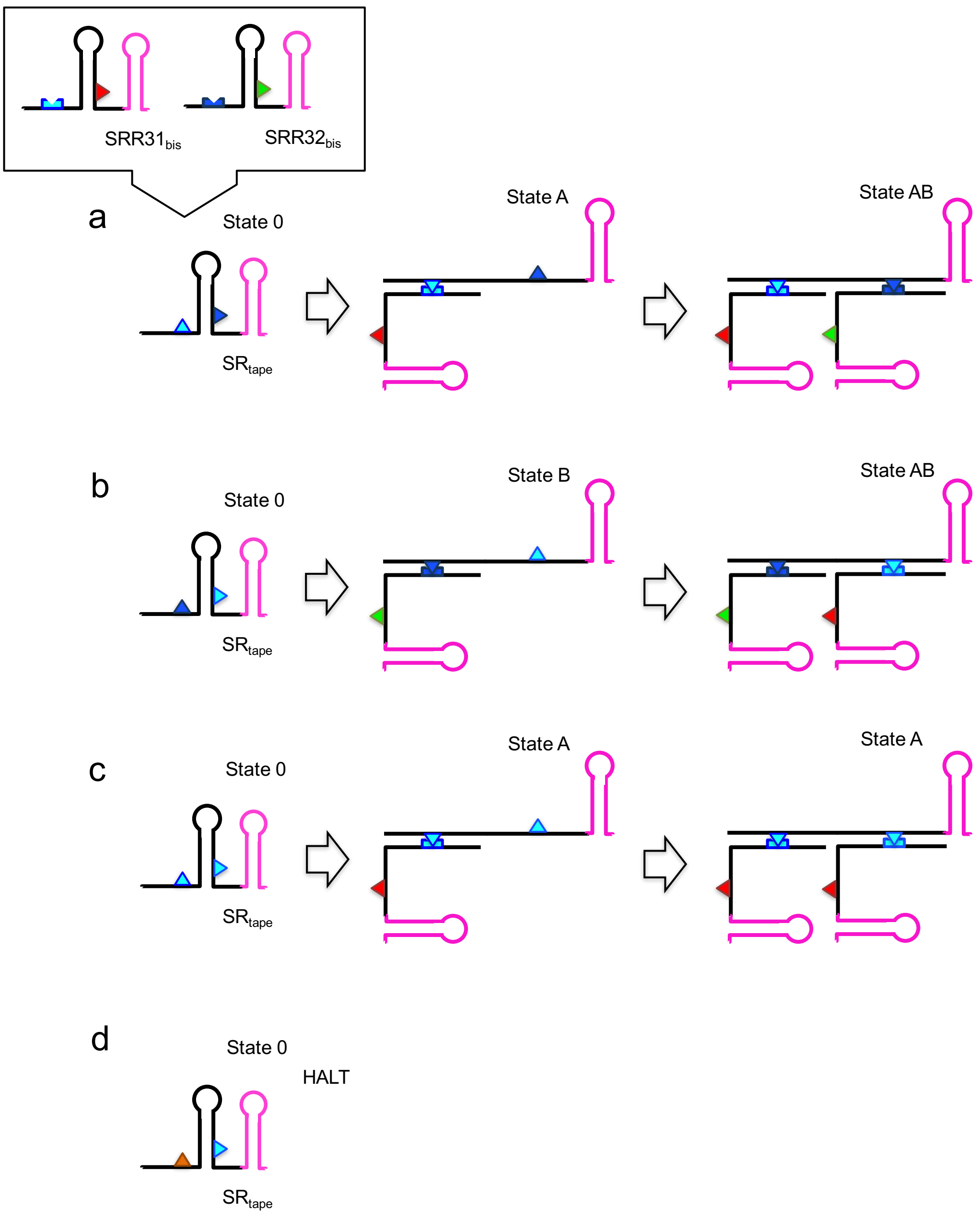

Current state	0	0	0	A	A	A	B	B	B
Symbol read	△ (light blue)	▲ (dark blue)	▲ (orange)	△ (light blue)	▲ (dark blue)	▲ (orange)	△ (light blue)	▲ (dark blue)	▲ (orange)
Symbol written	none	none	none	none	none	none	none	none	none
Move tape	left	left	HALT	left	left	HALT	left	left	HALT
New state	A	B	0	A	AB	A	AB	B	B

Figure S16. Illustration of how a Turing head implemented with two SRR molecules (here, SRR31$_{bis}$ and SRR32$_{bis}$) is able to read different tape molecules (appropriately designed). The Turing head also has a registry of the internal state implemented through SRRR molecules (here, SRRR31 and SRRR32), not shown for simplicity. On bottom, we show the transition state table of the machine.

Supplementary Tables

Table S1: Sequences of the RNA hybridization chain reactions designed in this work. Dot-bracket structures are also shown. The seed region for the interaction between the two sRNAs (SRR and SR) is shown in cyan. The seed region for the interaction between the sRNA complex and the 5' UTR (SRRR) is shown in red. In SRRR, the RBS is shown in yellow and the start codon in green. The transcription terminator T500 (efficiency > 90%) was used in SRR, shown in dark red, and the terminator TrrnC (efficiency > 90%) or B1002 (efficiency about 90%) in SR, shown in magenta (see ref. [27]).

System trigR2

>SRR2 (with T500)
UGGCGGCGCAGCGUCCGGCCCGCCUCACAUUUGCUCAACCAAAGCCCGCCGAAAGGCGGGCUUUUCU
GU
.(((((.((........)))))))................((((((((((....))))))))))...
..

>SR2 (with TrrnC)
ACUGGCGCGAAAUGUAGAGGUGGGCCGGACGAAUCCUUAGCGAAAGCUAAGGAUUUUUUUU
.(((((.((...........)).)))))..(((((((((((....))))))))))).....

>SRRR2
ACAUCGCAGGUUUCUGCCUGCCUGCGCCGCCACACAGUAGGAGAAAUUCGAUAUG
..((((..(((((((.(((((.((.(......).))))))))))))))))))...

System trigR1

>SRR1 (with T500)
AAUUUAGGCGGAGUUGGGUAGAGGACGCUGCUUGUACGCUCUCGUAUUGACGGCACCCGCGUCGAUG
UGAGGGACUUGGCAAAGCCCGCCGAAAGGCGGGCUUUUCUGU
....(((((((.(((........))).)))))))....((((((((((((((.......))))))))
))))))....((.((((((((((....)))))))))).))..

>SR1 (with B1002)
CAAGUCCGUGAAGUGUACGGGCAGCUUGAUAUUUCGACCCUACCAGUUGGAACUAUUAAUUUGGGAC
CAUUCAUAGUGGUUCCGAAGCGCAAAAAACCCCGCUUCGGCGGGGUUUUUUCGC
...(((((((.....)))))))...(((((((((((((.......))))))).))))))((((((((
((((...))))))))))))(((.((((((((((((....)))))))))))))))

>SRRR1
AGUUCCGACGGGUCUCCUCUUUCGACUCCGCUUGAAAGAGGAGGUUUGUCAUAUG
......(((((..((((((((((((......))))))))))))..))))).....

System trigR11

>SRR11 (with T500)
GGGAGGGUUGAUUGUGUGAGUCUGUCACAGUUCAGCGGACAAAGCCCGCCGAAAGGCGGGCUUUUCU
GU
......(((((...(((((.....)))))..))))).((.((((((((((....)))))))))))).
..

>SR11 (with TrrnC)
AACGUUGAUGCUGUGACAGAUUUAUGCGAGGCAUCCUUAGCGAAAGCUAAGGAUUUUUUUU
........((((.((.((......)))).))))((((((((....))))))))........

>SRRR11
CCUCGCAUAAUUUCACUUCUUCAAUCCUCCCGUUAAAGAGGAGAAAUUAUGAAUG
......((((((((.((((((.(((......))).)))))).)))))))).....

System trigR31

>SRR31 (with T500)
GGGUCUUAUCUUAUCUAUCUCGUUUAUCCCUGCAUACAGAAACAGACCAGAUAUGCAAUGAUAAACG
AGAACAAAGCCCGCCGAAAGGCGGCUUUUCUGU
................(((((((((((..(((((((.............).))))))..))))))))
)))..((((((((((....)))))))))).....

>SR31 (with TrrnC)
GGGACUGACUAUUCUGUGCAAUAGUCAGUAAAGCAGGGAUAAACGAGAUAGAUAAGAUAAGAUAGAA
AAUCCUUAGCGAAAGCUAAGGAUUUUUUUU
...((((((((((......))))))))))..................................((((
((((((((((....))))))))))))))..

>SRRR31
UCUCGUUUAUCAUUGUAUUUCCGGUUUGUUUCAACAGAGGAGAGAGACGAAAUGGAAGUACGACAC
............(((((((((((.((((((((...........)))))))).)))))))))))...

System trigR32

>SRR32 (with T500)
GGGUCACUUAAUCAUUUGUCGUCGUUUCUAUCUAUACAAGAACAGACCUCAUAUAGAAUGAAACGAC
GAAACCUGGCGGCAGCGCAAAAGAUGCGUAAACAAAGCCCGCCGAAAGGCGGCUUUUCUGU
(.(((.............((((((((((..((((((..((.......))..))))))..))))))))
))......((....))....))).)......((((((((((....))))))))))).....

>SR32 (with TrrnC)
GGGUCGAGUAGACAGAGCUGUCUACUCGAAUAAGAUAGAAACGACGACAAAUGAUUAAGUGAGAAAU
CCUUAGCGAAAGCUAAGGAUUUUUUUU
...(((((((((((....)))))))))))................................((((((
(((((((....)))))))))))))...

>SRRR32
ACGCAUUAUGUGCGUUGUCGCCCGUUUGUGUCUUUCAUUUCUAUAAUCAAAGGGAGUGGCAGUAUGU
AUAUGCGU
(((((((((((((..((((((((.((((...................)))).)).))))))))))))
).))))))

Controls on system trigR31

>SRR31Hfq (Hfq scaffold in gray)
GGGUCUUAUCUUAUCUAUCUCGUUUAUCCCUGCAUACAGAAACAGACCAGAUAUGCAAUGAUAAACG
AGAACGUCCCGCAAGGAUGCGGGUCUGUUUACCCCUAUUUCAACCGGCCGCCUCGCGGCCGGUUUUU
UUUU
((((............(((((((((((..(((((((.............).))))))..))))))))
)))..(.((((((....)))))).).....)))).......((((((((((...))))))))))...
....

>SRR31* (fusion; note that a 2-nt mutation was introduced to create a bulge in the large steam formed without affecting the free energies of interaction)
GGGACUGACUAUUCUGUGCAAUAGUCAGUAAAGCAGGGAUAAACGAGUAAGAUAAGAUAAGAUAGAA
AGGGUCUUAUCUUAUCUAUCUCGUUUAUCCCUGCAUACAGAAACAGACCAGAUAUGCAAUGAUAAAC
GAGAACAAAGCCCGCCGAAAGGCGGCUUUUCUGU
...((((((((((......))))))))))...(((((((((((((((..((((((((((((((....
...))))))))))))))..)))))))))))))))..((((..((...((....))...)).......
......((((((((((....))))))))))))))

>SRRb31 (interacting with SR31)
GGGUCUUAUCUUAUCUAUCUCGUUUAUCCCUGCAUACAGAAACAGAGGAGAUAUGCAAUGAUAAACG
AGAACCUGGCGGCAGCGCAAAAGAUGCGUAAA
(.(((((..........(((((((((((..(((((((............).))))))..))))))))
))).....(((....)))..))))).).....

Table S2: Strains and plasmids used in this work.

Strains or plasmids	Features	Ref.
E. coli DH5α	Commercial	Invitrogen
E. coli DH5α-Z1	Commercial (DH5α, *lacI*Q, PN25-*tetR*, SpR)	Clontech
E. coli MG1655-Z1	*lacI*Q, PN25-*tetR*, SpR	Gifted by M.B. Elowitz
ptrigR2	pSC101m ori, kanR, sfGFP-LAA	This work
ptrigR2St	pSC101m ori, kanR, sfGFP	This work
ptrigR1	pSC101m ori, kanR, sfGFP-LAA	This work
ptrigR11	pSC101m ori, kanR, sfGFP-LAA	This work
ptrigR11St	pSC101m ori, kanR, sfGFP	This work
ptrigR31	pSC101m ori, kanR, sfGFP-LAA	This work
ptrigR32	pSC101m ori, kanR, sfGFP-LAA	This work
pRAJ11	pUC ori, ampR-kanR, GFPmut3b	[1]
ptrigR31Hfq	pSC101m ori, kanR, sfGFP-LAA	This work
ptrigR31Fusion	pSC101m ori, kanR, sfGFP-LAA	This work
ptrigR11/2	sRNAs from system trigR11, 5' UTR from system trigR2 pSC101m ori, kanR, sfGFP-LAA	This work
ptrigR2/11	sRNAs from system trigR2, 5' UTR from system trigR11 pSC101m ori, kanR, sfGFP-LAA	This work
ptrigR31FFL	pUC ori, ampR, sfGFP-LAA (J23119:SRRb31)	This work

Table S3: Predicted values of the free energies of full hybridization (ΔG_1 and ΔG_2 for desired interactions, $\Delta\hat{G}_1$ and $\Delta\hat{G}_2$ for undesired ones) as well as toehold hybridization ($\Delta G_1^{\text{toehold}}$ and $\Delta G_2^{\text{toehold}}$ for desired interactions, $\Delta\hat{G}_1^{\text{toehold}}$ and $\Delta\hat{G}_2^{\text{toehold}}$ for undesired ones) for designer riboregulatory cascades. Also, predicted values of $\Delta G_{SRRR}^{\text{struct}}$ and $\Delta G_{SRRR*}^{\text{struct}}$ for those systems (calculated as previously done [2]). Values in Kcal/mol.

System	ΔG_1	$\Delta G_1^{\text{toehold}}$	ΔG_2	$\Delta G_2^{\text{toehold}}$
trigR31	-36.9	-17.9	-20.3	-11.2
trigR32	-30.1	-14.1	-18.6	-9.5
trigR1	-13.3	-4.8	-16.7	-9.5
trigR2	-23.9	-2.7	-19.3	-10.3
trigR11	-21.2	-2.3	-14.2	-10.3

$\Delta\hat{G}_1$	$\Delta\hat{G}_1^{\text{toehold}}$	$\Delta\hat{G}_2$	$\Delta\hat{G}_2^{\text{toehold}}$	$\Delta G_{SRRR}^{\text{struct}}$	$\Delta G_{SRRR*}^{\text{struct}}$
-15.6	-13.7	-20.9	0	1.2	0
-6.0	-3.4	-12.0	0	1.2	3.6
-5.6	0	-4.4	0	2.4	0
-3.1	0	-9.6	0	0	0
-8.6	0	-7.9	-10.3	2.4	0

Table S4. Cost of expressing the engineered sRNA systems in *E. coli*. The value of cell growth rate (h^{-1}), calculated by regressing OD_{600} with time during exponential phase (OD_{600} = 0.1 - 0.6), is shown for each induction condition. In brackets, the percentage of growth with respect to plain cells in the very same conditions.

	none	aTc	IPTG	aTc + IPTG
trigR31	0.2344 ± 0.0243 (97.3%)	0.2464 ± 0.0252 (102.3%)	0.1761 ± 0.0208 (73.1%)	0.1895 ± 0.0207 (78.7%)
trigR32	0.2665 ± 0.0096 (88.5%)	0.2728 ± 0.0017 (90.6%)	0.2201 ± 0.0042 (73.1%)	0.2233 ± 0.0041 (74.1%)
trigR1	0.3287 ± 0.0086 (99.9%)	0.3799 ± 0.0113 (115.4%)	0.3486 ± 0.0056 (105.9%)	0.3618 ± 0.0153 (109.9%)
trigR2	0.2975 ± 0.0202 (80.0%)	0.3106 ± 0.0216 (83.5%)	0.3033 ± 0.0203 (81.5%)	0.3246 ± 0.0152 (87.3%)
trigR11	0.2709 ± 0.0222 (69.7%)	0.2784 ± 0.0049 (71.6%)	0.2539 ± 0.0250 (65.3%)	0.2821 ± 0.0067 (72.6%)
trigR31FFL	0.2209 ± 0.0029 (86.3%)	0.2321 ± 0.0009 (90.6%)	0.1831 ± 0.0035 (71.5%)	0.1964 ± 0.0048 (76.7%)
plain cells	0.2408 ± 0.0022<br>0.3012 ± 0.0014<br>0.3291 ± 0.0029<br>0.3720 ± 0.0003<br>0.3886 ± 0.0068<br>0.2561 ± 0.0064	-	-	-

Table S5: Prediction of eventual off-target effect of the designed sRNAs using RNApredator [13]. Neighborhood of 90 nt before and 10 nt after the start codon (in *E. coli* K-12 MG1655). Essential genes bold-faced [28] (although the sRNAs do not hybridize with the RBSs of the essential genes targeted, expect SRR31 on *nusA*).

**Riboregulator**	**Potential target**
SRR2	*metH*
SR2	*mrcA, tusD, glpX, insH10, pyrF, arnA, clsC,* ***lptG****, yffL, entH*
SRR1	*rutC, adrA, ygeV, yfjR, melR*
SR1	*metB, rcsD, ykgE, gudX, ycbK*
SRR11	*ydfH, ttdT, yfiL, ydgD, melR, yegW, glpX, yicG,* ***hemH***
SR11	*dusB, phoB, iscU, yecE, phnD, sufC, yfcC, rimM*
SRR31	*insH11, quuD, rnc, ygaC,* ***rpsS****,* ***fbaA****,* ***nusA****, yhaM*
SR31	*yggI, quuD, mdtG, wcaE*
SRR32	*yggU, yafW, leuC*
SR32	*glgA, mprA, nfsB, yccS, rihA, phnP, ybjG, ybaY,* ***cdsA***

Table S6: Additional sequences for the theoretical design of an RNA-based computer. Dot-bracket structures are also shown.

```
>SRtape (with TrrnC)
GGGACUGACUAUUCUGUGCAAUAGUCAGUAAAGCAGGGAUAAACGAGAUAGAUAAGAUAAGAUAGAA
ACGUGGCGACGUUUCUAUCUUAUCUGUAAGUGACCAUCCUUAGCGAAAGCUAAGGAUUUUUUUU
...((((((((((......)))))))))).........................(((((((((((((
((((....))))))))))))))))).........(((((((((....))))))))).......

>SRR31bis (with T500)
ACGUUUCUAUCUUAUCUUAUCUAUCUCGUUUAUCCCUGCAUACAGAAACAGACCAGAUAUGCAAUGA
UAAACGAGAACAAAGCCCGCCGAAAGGCGGGCUUUUCUGU
.......................(((((((((((..((((((...............))))))..))
)))))))))..((((((((((....)))))))))).....

>SRR32bis (with T500)
UAUAAACGAGAUAGGUUAUUUAAAGAUAAGAUCGUCGCCUCGACUAUAAAAGAACAGACCUCAUAUA
GAAUGAAACGACGAAACCUGGCGGCAGCGCAAAAGAUGCGUAAACAAAGCCCGCCGAAAGGCGGGCU
UUUCUGU
......((...((((((..............((((((..(((.(((((..((.......))..))))
)..)))..)))))))))))).))...(((((.....)))))....((((((((((....))))))))
)).....
```

Supplementary References

[1] Rodrigo, G., Landrain, T.E., & Jaramillo, A. De novo automated design of small RNA circuits for engineering synthetic riboregulation in living cells. *Proc. Natl. Acad. Sci. USA* 109, 15271-15276 (2012).

[2] Rodrigo, G., Landrain, T.E., Majer, E., Daròs, J.-A., & Jaramillo, A. Full design automation of multi-state RNA devices to program gene expression using energy-based optimization. *PLoS Comput. Biol.* 9, e1003172 (2013).

[3] Hofacker, I.L., Fontana, W., Stadler, P.F., Bonhoeffer, L.S., Tacker, M., & Schuster, P. Fast folding and comparison of RNA secondary structures. *Monatsh. Chem.* 125, 167-188 (1994).

[4] Salis, H.M., Mirsky, E.A., & Voigt, C.A. Automated design of synthetic ribosome binding sites to control protein expression. *Nat. Biotechnol.* 27, 946-950 (2009).

[5] Green, A.A., Silver, P.A., Collins, J.J., & Yin, P. Toehold switches: de-novo-designed regulators of gene expression. *Cell* 159, 925-939 (2014).

[6] Schindelin, J., Arganda-Carreras, I., Frise, E., *et al.* Fiji: an open-source platform for biological-image analysis. *Nat. Methods* 9, 676-682 (2012).

[7] Phillips, R. Napoleon is in equilibrium. *Annu. Rev. Condens. Matter Phys.* 6, 85-111 (2015).

[8] Lutz, R., & Bujard, H. Independent and tight regulation of transcriptional units in Escherichia coli via the LacR/O, the TetR/O and AraC/I1-I2 regulatory elements. *Nucleic Acids Res.* 25, 1203-1210 (1997).

[9] Rodrigo, G., Majer, E., Prakash, S., Daros, J.A., Jaramillo, A., & Poyatos, J.F. Exploring the dynamics and mutational landscape of riboregulation with a minimal synthetic circuit in living cells. *Biophys. J.* 109, 1070-1076 (2015).

[10] Soper, T., Mandin, P., Majdalani, N., Gottesman, S., & Woodson, S.A. Positive regulation by small RNAs and the role of Hfq. *Proc. Natl. Acad. Sci. USA* 107, 9602-9607 (2010).

[11] Sakai, Y., Abe, K., Nakashima, S., Yoshida, W., Ferri, S., Sode, K., & Ikebukuro, K. Improving the gene-regulation ability of small RNAs by scaffold engineering in Escherichia coli. *ACS Synth. Biol.* 3, 152-162 (2014).

[12] Adamson, D.N., & Lim, H.N. Essential requirements for robust signaling in Hfq dependent small RNA networks. *PLoS Comput. Biol.* 7, e1002138 (2011).

[13] Eggenhofer, F., Tafer, H., Stadler, P.F., & Hofacker, I.L. RNApredator: Fast accessibility-based prediction of sRNA targets. *Nucleic Acids Res.* 39, W149-W154 (2011).

[14] Turing, A.M. On computable numbers, with an application to the Entscheidungsproblem. *Proc. Lond. Math. Soc.* 42, 230-265 (1936).

[15] Adleman, L.M. Molecular computation of solutions to combinatorial problems. *Science* 266, 1021-1024 (1994).

[16] Benenson, Y., Paz-Elizur, T., Adar, R., Keinan, E., Livneh, Z., & Shapiro, E. Programmable and autonomous computing machine made of biomolecules. *Nature* 414, 430-434 (2001).

[17] Shapiro, E., & Benenson, Y. Bringing DNA computers to life. *Sci. Am.* 294, 44-51 (2006).

[18] Amos, M., Axmann, I., Bluethgen, N., de la Cruz, F., Jaramillo, A., Rodriguez-Paton, A., & Simmel, F. Bacterial computing with engineered populations. *Phil. Trans. R. Soc. A* 373, 20140218 (2015).

[19] Regot, S., Macia, J., Conde, N., Furukawa, K., *et al*. Distributed biological computation with multicellular engineered networks. *Nature* 469, 207-211 (2011).

[20] Roquet, N., Soleimany, A.P., Ferris, A.C., Aaronson, S., & Lu, T.K. Synthetic recombinase-based state machines in living cells. *Science* 353, aad8559 (2016).

[21] Shipman, S.L., Nivala, J., Macklis, J.D., & Church, G.M. Molecular recordings by directed CRISPR spacer acquisition. *Science* 353, aaf1175 (2016).

[22] Liu, C.C., Qi, L., Lucks, J.B., Segall-Shapiro, T.H., Wang, D., Mutalik, V.K., & Arkin, A.P. An adaptor from translational to transcriptional control enables predictable assembly of complex regulation. *Nat. Methods* 9, 1088-1094 (2012).

[23] Qi, L.S., Larson, M.H., Gilbert, L.A., Doudna, J.A., Weissman, J.S., Arkin, A.P., & Lim, W.A. Repurposing CRISPR as an RNA-guided platform for sequence-specific control of gene expression. *Cell* 152, 1173-1183 (2013).

[24] Roth, A., Weinberg, Z., Chen, A.G.Y., Kim, P.B., Ames, T.D., & Breaker, R.R. A widespread self-cleaving ribozyme class is revealed by bioinformatics. *Nat. Chem. Biol.* 10, 56-60 (2013).

[25] Vogel, J., Bartels, V., Tang, T.H., Churakov, G., Slagter-Jager, J.G., Huttenhofer, A., & Wagner, E.G.H. RNomics in Escherichia coli detects new sRNA species and indicates parallel transcriptional output in bacteria. *Nucleic Acids Res.* 31, 6435-6443 (2003).

[26] Bennett, M.R., & Hasty, J. Microfluidic devices for measuring gene network dynamics in single cells. *Nat. Rev. Genet*. 10, 628-638 (2009).

[27] Rostain, W., Landrain, T.E., Rodrigo, G., & Jaramillo, A. Regulatory RNA design through evolutionary computation and strand displacement. *Methods Mol. Biol.* 1244, 63-78 (2015).

[28] Yamazaki, Y., Niki, H., & Kato, J. Profiling of Escherichia coli chromosome database. *Methods Mol. Biol*. 416, 385-389 (2008).
